# Insight into the formation of bismuth-tungsten carbonyl clusters

**DOI:** 10.1038/s42004-023-00905-6

**Published:** 2023-06-05

**Authors:** Katrin Beuthert, Benjamin Peerless, Stefanie Dehnen

**Affiliations:** Karlsruhe Institute of Technology (KIT), Institute of Nanotechnology (INT), 76021 Karlsruhe, Germany

**Keywords:** Chemical bonding, Solid-state chemistry, Coordination chemistry, Materials chemistry

## Abstract

Multimetallic clusters play a key role as models to doped metals, as candidates to new types of superatomic catalysts and as precursors to new multimetallic solids. Understanding formation pathways is an essential and necessary step forward in the development of cluster synthesis and research, yet remains considerably lacking owing to difficulty in identification of intermediates and the ill-defined nature of common starting materials. Here we show progress in this regard by investigating the reactivity of an intermetallic solid of nominal composition ‘K_5_Ga_2_Bi_4_’ with [W(cod)(CO)_4_] upon extraction with ethane-1,2-diamine (en) and 4,7,13,16,21,24-hexaoxa-1,10-diazabicyclo[8.8.8]hexacosane (crypt-222). Several polybismuthide intermediates and by-products were identified along the reaction pathway, ultimately forming the new polybismuthide salt [K(crypt-222)]_3_[µ:η^3^-Bi_3_{W(CO)_3_}_2_]∙en∙tol. DFT calculations revealed plausible reaction schemes for the transformations taking place in the reaction mixture providing insight into the complex reactivity of ‘K_5_Ga_2_Bi_4_’ on the basis of in situ generation of Bi_2_^2−^.

## Abstract (web summary)

Understanding the formation pathways of multimetallic clusters is essential to the progress of cluster research, but remains highly challenging. Here, time-dependent crystallization, mass spectrometry, and quantum chemical calculations are used to gain insight into the formation pathways of bismuth–tungsten carbonyl clusters.

## Introduction

Metal cluster molecules have been described as intermediary compounds between molecules and extended materials, or as model compounds for surface interactions involving the bulk phase especially when considering catalysis and their place in the “cluster-surface” analogy^1–7^. This is commonly employed with respect to transition metal carbonyl cluster compounds. Yet, despite their relevance and use, cluster molecules are in a position as exciting and fascinating compounds while their formation pathways are poorly understood. Given the vital role mechanistic detail applies to our greater knowledge of organic or organometallic chemistry, efforts to elucidate such pathways are essential^8,9^. The main drawback, in such efforts of cluster synthesis, is the limited understanding in how metal–metal bonds actually form in metal clusters, especially when there are several such bonds in a molecule. This lack of a retrosynthetic approach, ubiquitous in all fields of synthetic chemistry, significantly hinders this avenue of study. A starting point to solving these challenging problems lies in the diverse range of compounds that form upon the synthesis of a metal cluster compound. Zintl anions, homo- or heteroatomic anionic cluster molecules of the main group elements, are all-(semi)metal molecules with relatively flexible bonds, ideal as candidates for studying formation pathways in cluster synthesis^10–12^.

Intermetallic solid mixtures between alkali/alkaline earth metals and main group elements are the starting point for all known Zintl clusters^13^. Extraction of these mixtures with highly polar solvents, such as ethane-1,2-diamine (en) or liquid ammonia, in the presence of a sequestering agent, most commonly 4,7,13,16,21,24-hexaoxa-1,10-diazabicyclo[8.8.8]hexacosane (crypt-222), yields homoatomic or heteroatomic Zintl anions depending on the solid mixture used. When another reagent is added to the solid mixture, such as a transition metal (or f-block metal) complex, the Zintl anion can then be modified or expanded^14^. Realization of this reactivity opens up an incredible wealth of possibilities. The choice of transition metal and ligand sphere of the complex can have a profound effect on the outcome of the final product, as well as on the properties of the new cluster itself^15^. The functionalization and reactivity of Zintl clusters have been reviewed numerous times, and several new endeavours since these reviews, highlighting enhanced solubilization and catalysis for example, have been made^11,13–20^. What is becoming clear is that Zintl clusters—both naked and functionalised—are moving away from academic curiosities toward functional compounds in their own right^19,21–24^. With this, addressing the challenge of the formation pathways of these structurally complex and beautiful molecules is more apparent. This also addresses the efficiency of cluster formation or prediction. There are a few studies highlighting such pathways, and notably how difficult it is to model and predict the processes involved^10,12,25–27^.

**Fig. 1:**
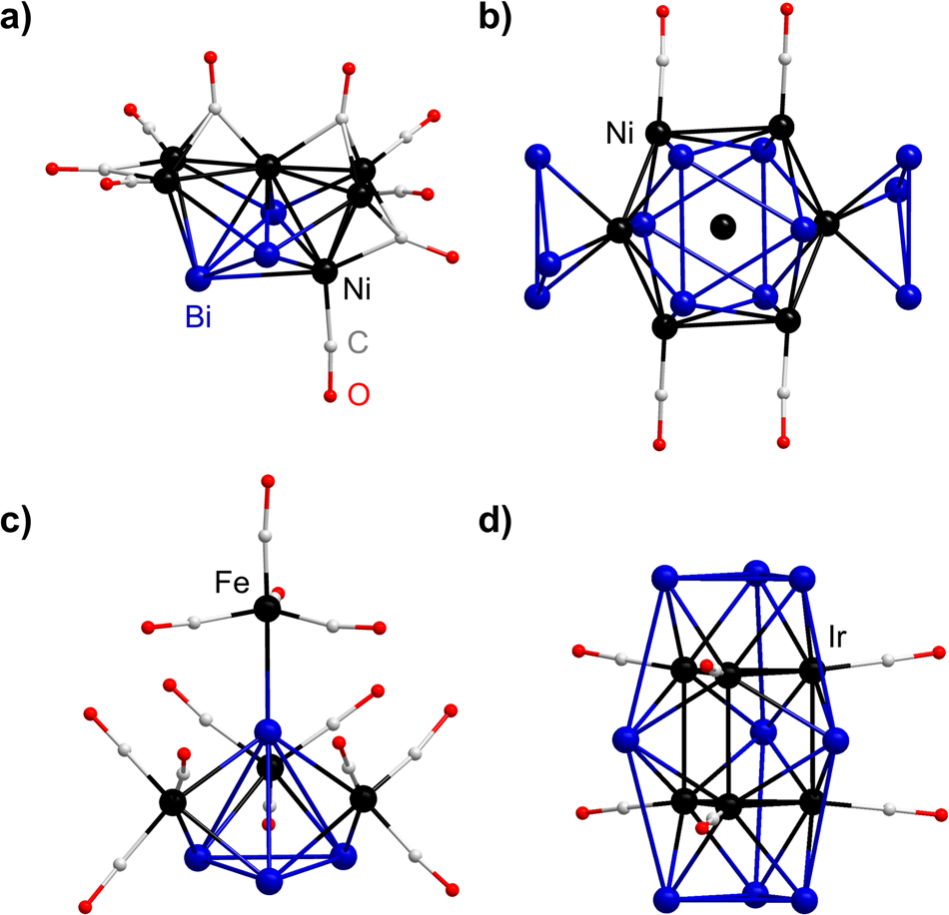
Examples of structures of known polybismuthide–metal clusters bearing CO ligands. **a** [Bi_3_Ni_6_(CO)_9_]^3–^^32^. **b** [Ni@{Bi_6_Ni_6_(CO)_8_}]^4–^^32^. **c** [Bi_4_Fe_4_(CO)_13_]^2–^^31^. **d** [(η^3^-Bi_3_)_2_(IrCO)_6_(µ^4^-Bi)_3_]^3–^^29^.

Therefore, we wanted to investigate cluster formation pathways in a systematic manner. The reactions between transition metal carbonyl clusters and Bi-based Zintl anions have been investigated a number of times in terms of the identification of products, but little insight into their formation. Seemingly, transition metal carbonyl complex fragments as a part of polybismuthide–metal cluster molecules, [Bi_x_{(M(CO)_y_)}_z_]^q−^, that can be described as having fewer than 12 electrons, like [M(CO)]^+^ (M = Co, Rh, Ir) with 10 electrons, yield cluster motifs typical for electron-rich species (thus, not consistent with deltahedral cage types)^28–30^. In contrast, those with 12 electrons, [Ni(CO)], [Cr(CO)_3_] and [Mo(CO)_3_] for example, do typically possess deltahedral (*closo*-)structures, as typical for electron-deficient cages^31–36^. Selected examples of known polybismuthide–metal clusters with CO ligands are shown in Fig. 1.

Another factor in the various structures observed is the flexibility of polybismuthide substructures where the {Bi_n_} fragment can range with values of *n* = 1–6 forming chains, rings, butterfly structures and even prisms and crowns. With such versatility in both the polybismuthide and transition metal complex fragment possible, insight into their formation would provide extensive information into cluster formation in general and even the potential of cluster design and fabrication on demand. Here, we report on the reactivity of a Bi intermetallic solid mixture, as a source for Bi-based Zintl anions, with group 6 transition metal carbonyl complexes. By isolation of several intermediate and by-product compounds along the reaction path accompanied by comprehensive quantum chemical studies, we have devised a pathway for the formation of complex polybismuthide–transition metal carbonyl clusters in an effort to elucidate key processes which can occur during cluster formation.

## Results and discussion

### Synthesis and structures

The intermetallic solid with the nominal composition “K_5_Ga_2_Bi_4_” has been used successfully multiple times as a source of polybismuthides and Bi-based Zintl clusters^10,37–41^. It has been suggested that the negatively charged Ga atoms in this mixture can act as a source of electrons by the release of elemental Ga to afford polybismuthides in the reaction mixture. This includes those that have not, or cannot, be isolated themselves, like Bi_3_^q−^, which has been observed in mass spectrometric measurements and long been postulated as an intermediate towards larger polybismuthides. To date, the only Ga- and Bi-containing anionic clusters reported are (GaBi_3_)^2−^(see ref. ^41^), (Ga_2_Bi_16_)^4−^, [Bi@Ga_8_(Bi_2_)_6_]^q−^ (*q* = 3 or 5)^10^ and [Sm@Ga_2_HBi_11_]^3−^(see ref. ^40^), with only the former being discussed to form upon extraction of the intermetallic solid alone; the other clusters requiring an oxidizing agent added during the extraction process, as their overall negative charge per atom is lower than that in the precursor material. Nevertheless, with several uncertainties it is clear the reactivity of “K_5_Ga_2_Bi_4_” is complex and requires further investigation given the effective application of this solid in Zintl cluster syntheses. We have therefore undertaken a systematic study into cluster formation reactions using “K_5_Ga_2_Bi_4_“ and one selected transition metal complex. On the combination of a 1:1:5 mixture of “K_5_Ga_2_Bi_4_”, [W(cod)(CO)_4_] (cod = 1,5-cyclooctadiene) and crypt-222 in en, a dark green suspension forms. Depending on the time which the suspension is allowed to stir different products are obtained. The reaction was effectively stopped by filtering the supernatant solution off the reactive intermetallic solid and subsequently layering it with toluene for crystallization (see experimental section for more detailed description). All isolated crystals’ constitution was additionally confirmed by micro-X-ray fluorescence spectroscopy (µ-XFS). Efforts to obtain more in situ data by optical spectroscopy or mass spectrometry was unsuccessful, therefore the following results are a combination of structural data and in-depth quantum chemical calculations.

**Fig. 2:**
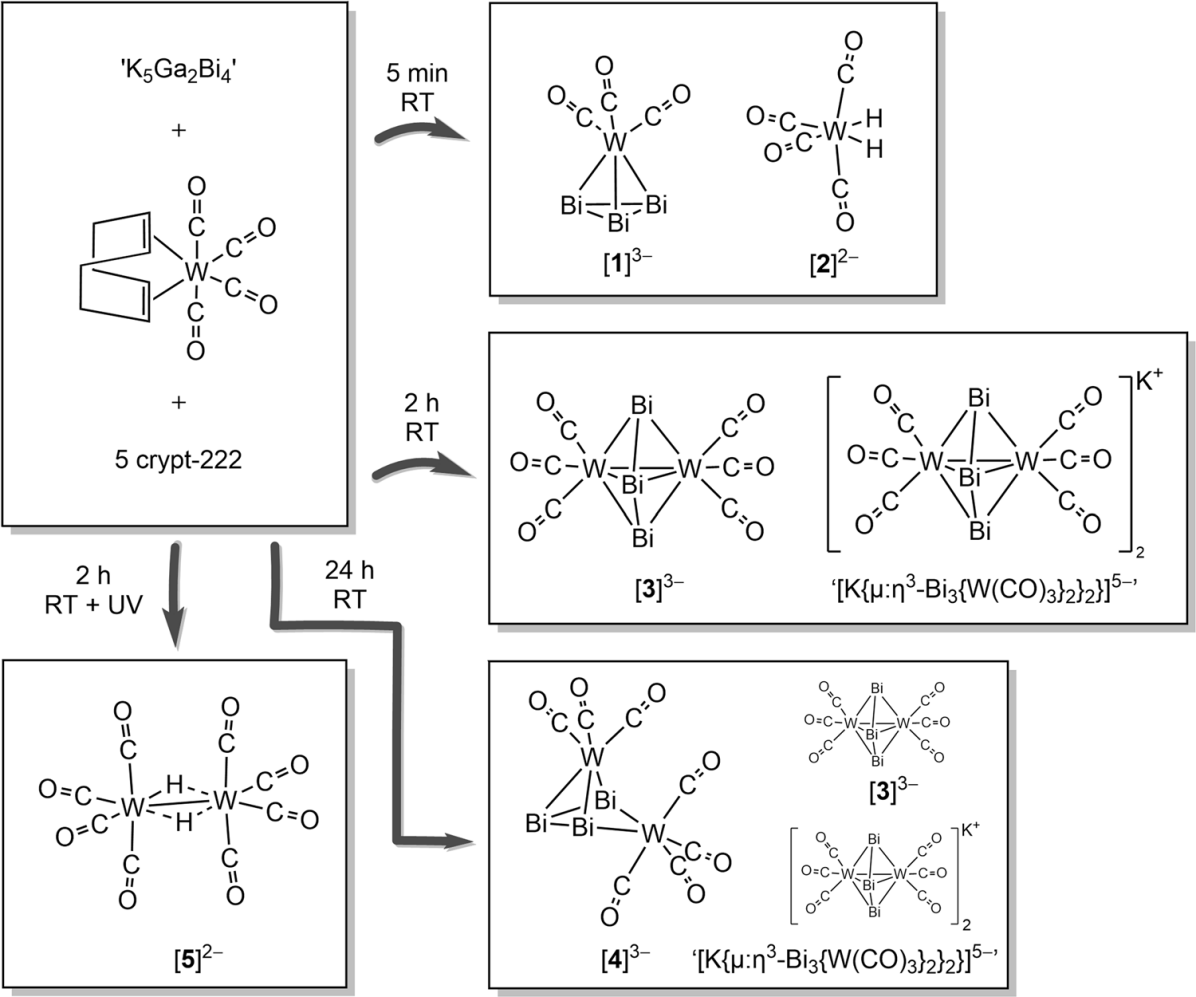
Sequential formation of the cluster compounds and complexes discussed in this work. The starting materials are shown in the top left corner, with all subsequent steps indicated by arrows.

After a reaction time of five minutes, crystals of two habits had formed: black, block crystals and green, plate crystals. Photographs of single crystals and reaction solutions are shown in Supplementary Fig. 1. The crystals were identified as [K(crypt-222)]_3_[η^3^-Bi_3_W(CO)_3_]∙3en∙3tol ([K(crypt-222)]_3_**1**∙3en∙3tol) and [K(crypt-222)]_2_[W(CO)_4_(H)_2_]∙en ([K(crypt-222)]_2_**2**∙en) by single-crystal X-ray diffraction, respectively. When the reaction time was prolonged to two hours, neither [K(crypt-222)]_3_**1**∙3en∙3tol or [K(crypt-222)]_2_**2**∙en was observed. In fact, two new compounds crystallized instead, [K(crypt-222)]_3_[µ:η^3^-Bi_3_{W(CO)_3_}_2_]∙en∙tol ([K(crypt-222)]_3_**3**∙en∙tol) and “[K(crypt-222)]_5_[K{µ:η^3^-Bi_3_{W(CO)_3_}_2_}_2_}]∙2en” as black needle and dark red needle crystals, respectively. Both [K(crypt-222)]_3_**3**∙en∙tol and “[K(crypt-222)]_5_[K{µ:η^3^-Bi_3_{W(CO)_3_}_2_}_2_}]∙2en” contain the same [µ:η^3^-Bi_3_{W(CO)_3_}_2_]^3–^ unit, which can be viewed as an addition of a {W(CO)_3_} fragment to anion [**1**]^3−^, though evidence suggests that it is more likely that these two compounds form upon addition of a {W(CO)_4_} unit to anion [**1**]^3−^ first. Upon increasing the reaction time to 24 h, ([K(crypt-222)]_3_**3**∙en∙tol and “[K(crypt-222)]_5_[K{µ:η^3^-Bi_3_{W(CO)_3_}_2_}_2_}]∙2en” again crystallize from the solution, however, a third compound also crystallizes alongside, [K(crypt-222)]_3_[µ^2^:η^3^-Bi_3_{W(CO)_3_W(CO)_4_}]∙3en∙tol ([K(crypt-222)]_3_**4**∙3en∙tol), as black, block crystals. One additional experiment performed was the exposure of the reaction mixture to UV-A radiation. No bismuth-containing product was obtained, only [K(crypt)]_2_[W_2_CO_8_(µ-H_2_)] ∙ 0.5tol ([K(crypt-222)]_2_**5** ∙ 0.5tol) was observed from a dark-brown solution as yellow plate-shaped crystals. Mass spectrometric measurements of reaction solutions and samples made from isolated crystals were unsuccessful. The observations made after different reaction times are summarized in Fig. 2.

**Fig. 3:**
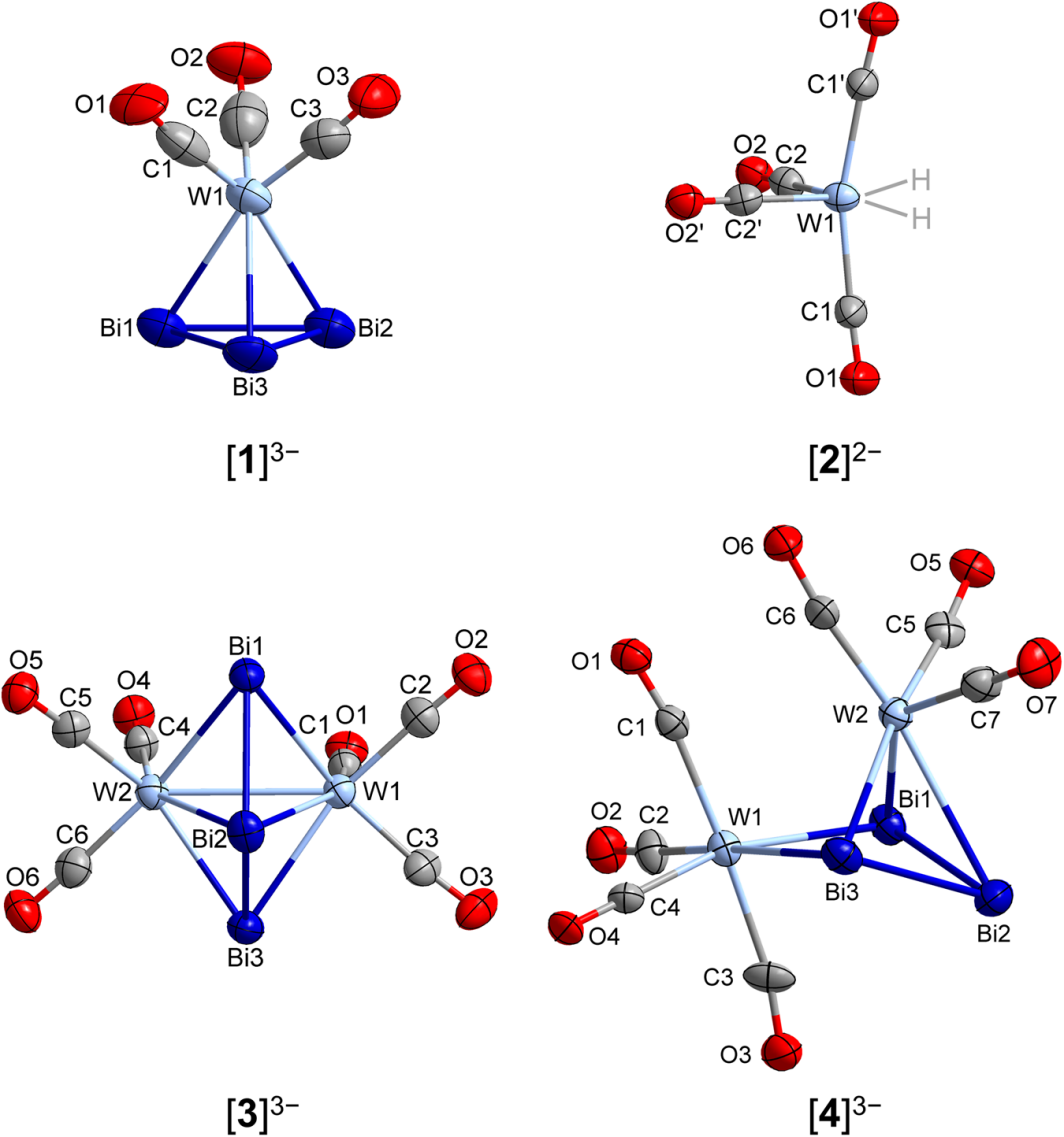
Molecular structures of the anions [η^3^-Bi_3_W(CO)_3_]^3–^ ([**1**]^3–^), [W(CO)_4_(H)_2_]^2–^ ([**2**]^2–^), [µ:η^3^-Bi_3_{W(CO)_3_}_2_]^3–^ ([**3**]^3–^), and [µ^2^:η^3^-Bi_3_{W(CO)_3_W(CO)_4_}]^3–^ ([**4**]^3–^). The structures were determined by means of single-crystal X-ray diffraction of the corresponding salts. Note that the H atoms in [**2**]^2–^ could not be refined from the difference Fourier map, nor could they be affixed to the structure; their positions are therefore indicated schematically. However, the presence of H atoms at these coordination sites was unambiguously confirmed by quantum chemical calculations of the structure of the {W(CO)_4_}^2–^ fragment with and without the H atoms (see below).

All six products were characterized by single-crystal X-ray crystallography. Crystallographic details are provided in Supplementary Data 1–5. Compounds [K(crypt-222)]_3_**1**∙3en∙3tol and “[K(crypt-222)]_5_[K{µ:η^3^-Bi_3_{W(CO)_3_}_2_}_2_}]∙2en” are the W analogs of previously described compounds of the lighter congeners Cr and Mo^36,42^, and due to disorder and poor data quality will not be discussed in detail beyond their connectivity. [**1**]^3−^ has a three-legged piano stool structure with a {Bi_3_} triangle capping the top, consistent with the previously reported structures containing Cr and Mo. The anion “[K{µ:η^3^-Bi_3_{W(CO)_3_}_2_}_2_}]^5−^” has the same structure as the anion in [K(crypt-222)]_5_[K{Bi_3_M_2_(CO)_6_}_2_] (M = Cr and Mo)^36^; it is given here in inverted commas, as the data sets collected from the crystals did not allow for a proper refinement of the entire crystal structure, although it can be assumed to be homologous to the lighter homolog. Compounds [K(crypt-222)]_2_**2**∙en, [K(crypt-222)]_3_**3**∙en∙tol, [K(crypt-222)]_3_**4**∙3en∙tol and [K(crypt-222)]_2_**5** ∙ 0.5tol are all newly identified structures. Anion [**3**]^3−^ has the same µ^3^-“ozone-like” {Bi_3_} structure bridging between two {W(CO)_3_} fragments observed in the structures of the [K(crypt-222)]_5_[K{Bi_3_M_2_(CO)_6_}_2_] series (M = Cr, Mo, W). The Bi−Bi bond lengths of the {Bi_3_} are slightly longer in the W case (3.015(1)/3.016(1) Å vs 2.956/2.954 for Cr vs 2.987/2.987 for Mo), and consistent with a bond length larger than a Bi=Bi double bond. In addition, the W−Bi bond lengths are also slightly longer compared to the Cr and Mo examples, as expected for the larger metal atom, and follow the same trend that the W−Bi_(***Bi***Bi***Bi***)_ bond lengths (2.831(1)-2.860(1) Å) are shorter than W−Bi_(Bi***Bi***Bi)_ (3.115(1)-3.120(1) Å). The presence of this {Bi_3_} moiety locks the geometry about the W atoms in place, forcing the three CO ligands on each metal atom into an eclipsed conformation. Anion [**4**]^3−^ is an interesting extension to the coordination chemistry of {Bi_3_} fragments. In the molecular structure, the {Bi_3_} unit does not bridge symmetrically between the two chemically different W atoms. One W atom, that belongs to the {W(CO)_3_} fragment, is bonded to the three Bi atoms in an η^3^-fashion whilst the W atom of the {W(CO)_4_} fragment is only bonded to two of the Bi atoms affording six bonds to each W atom. This η^2^-coordination, in fact, effectively forms a puckered four-membered {WBi_3_} ring, which coordinates through the three Bi atoms to {W(CO)_3_} giving a geometry consistent with a three-legged piano stool complex for this W atom. A result of the different coordination mode in {W_2_Bi_3_} leads to a slight decrease in the Bi−Bi bond length (2.961(1) and 2.987(1) Å) and a narrower Bi−Bi−Bi bond angle (90.34(3)° vs. 96.26(2)°) compared to that of anion [**3**]^3−^. The {(CO)_4_W}−Bi bond lengths are similar to one another (2.968(1) and 2.994(1) Å) yet slightly longer than {(CO)_3_W}−µBi (2.839(1) and 2.883(1) Å), the other {(CO)_3_W}−Bi bond length is the longest in the molecule (3.077(1) Å). Three of the CO ligands in the {W(CO)_4_} fragment are in an eclipsed arrangement with respect to the CO ligands of the {W(CO)_3_} fragment, and the fourth CO of the {W(CO)_4_} lies in the same plane as the non-bridging Bi atom. The molecular structures of the products are shown in Fig. 3.

At first glance, it can be thought that [**4**]^3−^ is the product of a reaction between [**1**]^3−^and [W(cod)(CO)_4_] starting material with concurrent loss of cod ligand. However, it then appears that the last compound to be experimentally observed is a compound that should be consumed at an earlier stage, as it is feasible that [**3**]^3−^ and “[K{µ:η^3^-Bi_3_{W(CO)_3_}_2_}_2_}]^5−^” could be generated by loss of CO from [**4**]^3−^. This idea is further supported that after allowing the mixture to stir for 48 h only [K(crypt-222)]_3_**3**∙en∙tol was isolated, additionally by stirring the mixture for 2 h at 60 °C the same outcome was observed. Though there is no quantitative kinetic evidence, only a qualitative description can be formed, suggesting that both the formation of the anionic complexes [**3**]^3−^ and [**4**]^3−^ are similarly slow processes. As mentioned above, irradiation by light did not yield another Bi-based compound, but led to the formation of the dinuclear W complex [**5**]^2–^.

### Quantum chemical calculations and formation pathway

To provide further insight into the reaction cascade, DFT calculations on all anionic molecules were performed, using the Turbomole program suite^43^. We employed the TPSS functional^44^ and dhf-TZVP basis sets^45^ including pseudo potentials ECP-60 for W and Bi atoms^46^. Negative charges of the cluster anions were compensated by application of the conductor-like screening model (COSMO) with ε = infinity, rsolv = 1.3^47^. All optimized structures were consistent with the observed crystal structures (discussed below), and also helped elucidate the structure of [K(crypt-222)_2_]**2**∙en, as it was not possible to assign the hydrogen atom positions to the heavy W atoms accurately from crystallographic data alone. Of the minimum structures calculated for [W(CO)_4_]^2−^ and [W(CO)_4_(H)_2_]^2−^, only the latter is consistent with the observed molecular structure from the crystallographic data, with ~90° angles between the CO ligands, yet two *trans*-CO ligands angled slightly towards one another in the direction of the two hydrogen atoms and *C*_2v_ symmetry. [W(CO)_4_]^2−^, a 16-electron complex, has *C*_3v_ symmetry with three “planar” CO groups in 120° angles puckered up slightly away from the plane toward the CO along the rotational axis not conforming with the experimental structure.

Using the optimized energies, a plausible reaction scheme has been devised to account for the observed products and potential by-products along with identifying the most likely starting materials. This last point is drawn from the fact that “K_5_Ga_2_Bi_4_” is an ill-defined solid mixture, and upon extraction with crypt-222 and en multiple polybismuthides are present in solution.

The considered polybismuthides in this study, which were anticipated to form in situ from the reactant “K_5_Ga_2_Bi_4_”, were a Bi_2_^2−^ dumbbell^48^, a planar four-membered ring Bi_4_^2−^^49^, and the *pseudo*-tetrahedron (GaBi_3_)^2−^^41^, all compounds that have been observed previously on extraction of bismuth intermetallics. In addition, the hypothetical species Bi_3_^2−^ and Bi_3_^3–^ were also considered—the first (open-shell) anion can be viewed as what is formed from (GaBi_3_)^2−^ upon release of a Ga^0^ atom, and the latter is the 6π-aromatic expansion of Bi_3_^+^ (isoelectronic to C_3_H_3_)^+^). For the by-products, the release of cod and CO from [W(cod)(CO)_4_] is a reasonable assumption, however, an issue quickly arises when balancing the charges of each side of the reaction. This issue is common in Zintl chemistry and is often explained by the reduction of en to form (H_2_NCH_2_CH_2_NH)^−^ and production of H_2_, though this anion has never been directly observed^27,50,51^.

**Fig. 4:**
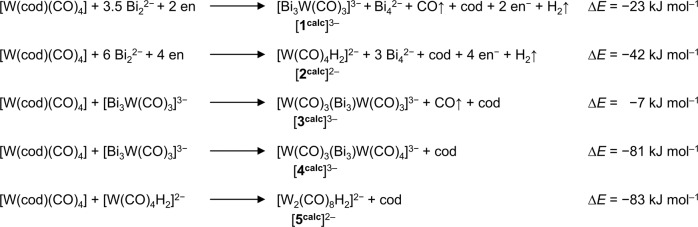
Reaction schemes and reactions energies for the formation of anions [1^calc^]^3–^, [2^calc^]^2–^, [3^calc^]^3–^, [4^calc^]^3–^, and [5^calc^]^2–^. Reaction energies are calculated from the total energies of the molecules obtained at the TPSS/dhf-TZVP level of theory. An arrow beside a molecule indicates its release as gas during the reaction— in agreement with the experimental observations of gas formation. Note that the first two reactions likely take place simultaneously but are given separately for the sake of clarity.

Balanced reaction schemes taking all these points into consideration were devised for the four starting polybismuthides or (GaBi_3_)^2–^. Of these, only the scheme involving Bi_2_^2−^ as starting material was calculated to have a favorable reaction energy (see Supporting Information for other reaction schemes). The reaction steps, and thus, the chemical relationship of calculated starting materials with the cluster anions [**1**^**calc**^]^3–^, [**2**^**calc**^]^2–^, [**3**^**calc**^]^3–^, [**4**^**calc**^]^3–^, and [**5**^**calc**^]^2–^ obtained from them are illustrated in Fig. 4.

We suggest that anions [**1**]^3−^ and [**2**]^2−^ form independently of one another from the reaction between Bi_2_^2−^ and [W(cod)(CO)_4_], both generating Bi_4_^2−^ as an additional by-product to the ones mentioned above. These processes are exoenergetic by −23 kJ mol^−1^ and −42 kJ mol^−1^, respectively. From anion [**1**]^3−^, the reaction cascade continues by addition of another [W(cod)(CO)_4_] with simple loss of (cod) to give anion [**4**]^3−^. This process has a calculated reaction energy of −81 kJ mol^−1^. Anion [**3**]^3−^ is then formed upon loss of a CO ligand from [**4**]^3−^ (anion “[K{µ:η^3^-Bi_3_{W(CO)_3_}_2_}_2_}]^5−^” was not considered for the reaction cascade as dissolved in a coordinating solvent such as en renders it unlikely to be present in solution), though this final step has a relatively small energy change at −7 kJ mol^−1^ for the calculated species. Such a low value offers a possible indication as to the observed long reaction time for complete conversion to anion [**3**]^3−^. Overall, there is a clear indication that the cluster anions are snapshots along the reaction pathway to the final product, [K(crypt-222)]_3_**3**∙en∙tol, starting from Bi_2_^2−^. The role of Ga remains uncertain, as is whether [**2**]^2−^ is involved or is simply a competing side reaction. Attempts involving KBi_2_ and K_3_Bi_2_ in place of K_5_Ga_2_Bi_4_ were unsuccessful so far, implying that Ga may have an influence if only to aid in the generation of Bi_2_^2−^ in solution as these solids are not reliable sources of Bi_2_^2−^ (another indication that Bi_2_^2−^ is most likely the active reagent). We would like to add that we cannot exclude that additional processes, like the formation of ion pairs, play a role in the mechanism. However, as we do not have any direct experimental evidence for such features, we did not consider them here.

The identity of [K(crypt-222)]_2_**2**∙en was, as alluded to earlier, confirmed by DFT calculations to contain the [W(CO)_4_(H)_2_]^2−^ dianion, as only the connectivity of the carbonyl ligands, yet not their relative orientation in the coordination sphere of the W atom, can be assigned with the experimental data alone. In the molecular structure there is a clear bending of the two *trans*-CO ligands (∠_C−W−C_ = 163.9(3)°) towards where the W − H bonds would be situated. The observed disphenoidal geometry consists of two chemically equivalent pairs of CO ligands. All W−C bonds are very close in length, though the two *trans*-CO ligands are slightly longer (1.996(5) and 1.967(4) Å). Reversely, the C−O bond lengths are longer in the *cis*-CO ligands, however, are effectively the same within the ESD. The *cis*-C−W−C bond angles, while being close to a rectangular arrangement, all slightly exceed 90°, compensated certainly by the bond angles involving the two unassignable hydrogen atoms. Anion [**5**]^2−^ in [K(crypt-222)]_2_**5**∙0.5tol is made up of two {W(CO)_4_} fragments, which are correspondingly bridged by two hydrogen atoms, leading to octahedral geometries about both W atoms again. The W^…^W distance is 3.021(1) Å. Similarly to [**2**]^2−^, the W−C bond lengths of the *trans*-CO ligands are slightly longer than the *cis*-CO ligands (1.978(8) and 1.929(8) Å). Unlike in [**2**]^2−^, the C−W−C ligands are consistent with a rather perfectly octahedral geometry of approximately 90 and 180°. It is reasonable to assume that [**5**]^2−^ is formed by the oxidative coupling of two [**2**]^2−^ upon release of H_2_ initiated by UV-light.

To summarize, several compounds which represent snapshots along the reaction pathway from the reaction between “K_5_Ga_2_Bi_4_” and [W(cod)(CO)_4_] in en have been identified, with the final product being the salt of the [µ:η^3^-Bi_3_{W(CO)_3_}_2_]^3−^ anion. From our observations, and supported by DFT calculations, balanced reaction schemes have been described for these processes which has shed light onto the reactivity of “K_5_Ga_2_Bi_4_” for the first time in such detail. The still ill-defined intermetallic solid has been proven as a source for a variety of polybismuthides or (GaBi_3_)^2–^, however, according to our findings, it is Bi_2_^2−^ that fits best as the reactive component of the extracted solution. The versatility of this simple molecule provides an excellent starting point for the growth of polybismuthide structures. With every study, the understanding and prediction of polybismuthide synthesis is becoming easier yet we are still only scratching the surface into the vast potential of these compounds.

## Methods

### General methods

All syntheses were performed under the exclusion of air and moisture using standard Schlenk or glovebox techniques. Ethane-1,2-diamine (en) was distilled from CaH_2_ and stored over 3 Å molecular sieves. Toluene (tol, Acros Organics, 99%) was distilled from sodium and stored over 3 Å molecular sieves. Kryptofix® 222 (crypt-222, Sigma Aldrich) was dried under vacuum for at least 18 h. [W(cod)(CO)_4_] was purchased from Sigma Aldrich. “K_5_Ga_2_Bi_4_” was prepared by stoichiometric fusion of the elements at 600 °C for 7 days in a niobium tube, sealed within an evacuated silica ampoule. For the standard procedure, “K_5_Ga_2_Bi_4_” (0.1 g, 85.4 μmol), [W(cod)(CO)_4_] (0.037 g, 85.4 μmol, 1 eq), and crypt-222 (0.161 g, 431.1 μmol, 5 eq) were dissolved in 4 ml en in a Schlenk tube and stirred at room temperature for a certain amount of time. Photographs of as-prepared crystal are shown in Supplementary Fig. 1.

### Synthesis of ([K(crypt-222)]_3_1∙3en∙3tol and ([K(crypt-222)]_2_2∙en

Following the standard procedure, the dark green reaction solution was filtered through a glass frit after five minutes. The dark green filtrate was layered with 5 ml tol for crystallization. After 1 week black, block crystals of ([K(crypt-222)]_3_**1**∙3en∙3tol and green plate crystals of ([K(crypt-222)]_2_**2**∙en had formed.

### Synthesis of ([K(crypt-222)]_3_3∙en∙tol and “[K(crypt-222)]_5_[K{µ:η^3^-Bi_3_{W(CO)_3_}_2_}_2_}]∙2en”

By extending the reaction time of the standard procedure to 2 h, no change in the color of the reaction solution was observed. The dark green reaction solution was layered with 5 ml tol. After 3 days, dark red needle crystals of ([K(crypt-222)]_3_**3**∙en∙tol and after one week black needle crystals of “[K(crypt-222)]_5_[K{µ:η^3^-Bi_3_{W(CO)_3_}_2_}_2_}]∙2en” had formed. The reaction could be repeated at 60 °C with the same results.

### Synthesis of ([K(crypt-222)]_3_4∙3en∙tol

The standard procedure was carried out with a reaction time of 24 h. The dark green reaction solution was filtered through a glass frit and layered with 5 ml tol. After 3 days, crystals in the form of black blocks, identified as ([K(crypt-222)]_3_**4**∙3en∙tol were obtained, beside crystals of [K(crypt-222)]_3_**3**∙en∙tol, and after one week also crystals of “[K(crypt-222)]_5_[K{µ:η^3^-Bi_3_{W(CO)_3_}_2_}_2_}]∙2en”.

### Synthesis of [K(crypt-222)]_2_5 ∙ 0.5tol

“K_5_Ga_2_Bi_4_” (0.1 g, 85.4 μmol), [W(cod)(CO)4] (0.037 g, 85.4 μmol, 1 eq), and crypt-222 (0.161 g, 431.1 μmol, 5 eq) were dissolved in 4 ml en a Schlenk tube and stirred for 2 h at room temperature and irradiated with UV-A light by application of an ULTRA-VITALUX UV-A lamp (Osram). The dark-brown solution was filtered using a glass frit. The brown filtrate was layered with 5 ml of tol. After 2 days, golden yellow crystals of [K(crypt-222)]_2_**5** ∙ 0.5tol were obtained in the form of thin platelets, additionally black cubes could be obtained. The structure of the cubes could not be analyzed.

### Micro-X-ray fluorescence spectroscopy (µ-XFS)

All µ-XFS measurements were performed on single-crystal samples with a Bruker M4 Tornado, equipped with an Rh-target X-ray tube, poly capillary optics and a Si drift detector. The emitted fluorescence photons are detected with an acquisition time of 180 s. Quantification of the elements is achieved through deconvolution of the spectra. Results are shown in Supplementary Table 1 and Supplementary Figs. 2–6, and it should be noted that for samples of Zintl cluster salts, the determination of the K content typically shows some deviations.

### Electrospray ionization mass spectrometry (ESI-MS)

ESI mass spectra were recorded with a Thermo Fischer Scientific Finnigan LTQ-FT spectrometer in the negative ion mode. In situ samples were taken from the reaction mixture and used directly. The solutions were injected into the spectrometer with gastight 250 µL Hamilton syringes by syringe pump infusion. All capillaries within the system were washed with dry en 2 h before and at least 5 min in between measurements to avoid decomposition reactions and consequent clogging. The following ESI parameters were used: Spray Voltage: 3.6 kV, Capillary Temp: 290 °C, Capillary Voltage: −20 kV, Tube lens Voltage: −121.75 kV, Sheath Gas: 45, Sweep Gas: 0, Auxiliary Gas: 40. The overview spectrum is given in Supplementary Fig. 7. Assignable signals are summarized in Supplementary Table 2, with the corresponding high-resolution spectra shown in Supplementary Figs. 8–12.

### Single-crystal X-ray diffraction data

The data for the X-ray structural analyses were collected at *T* = 100.0 K with Mo-Kα-radiation (*λ* = 0.71073 Å) on area detector systems Stoe IPDS/2T for *X, Y*, and *Z* and with Cu-K_α_-radiation (*λ* = 1.54186 Å) for *X, Y, Z* on an area detector system Stoe StadiVari. The structures were solved by methods of SHELXT from SHELXL-2018/136, and refined by full-matrix least-squares methods against *F*^2^ with the SHELXL^52^ program using Olex^2^ v1.3.0 software^53^. All hydrogen atoms were kept riding on calculated positions with isotropic displacement parameters *U* = 1.2 *U*_eq_ of the bonding partners. The crystal data and experimental parameters of the structure determination are collected in Supplementary Tables 3–8. Supplementary structural images are shown in Supplementary Figs. 13–26. Figures were created with Diamond 4^54^.

### Infrared (IR) spectroscopy

The sample was measured as solid on a BRUKER Alpha-P ATR-FT-IR spectrometer with a diamond ATR probe head in an inert gas atmosphere. The spectra were evaluated using the program OPUS 6.5. The position of the absorption bands is given in wavenumbers *ν* (unit cm^−1^). Measurements were made in the range 4000–500 cm^−1^. The experimental spectrum is given in Supplementary Fig. 27, and calculated IR spectra are shown in Supplementary Figs. 28–32.

### Quantum chemical calculations

DFT calculations were done with the program system TURBOMOLE^43^ using the TPSS functional^44^ and employing dhf-TZVP basis sets^45^, for Bi and W, Dirac–Fock in combination with effective core potentials ECP-60^46^. The conductor-like screening model (COSMO; ε = infinity, rsolv = 1.3) was used for charge compensation of the anionic molecules^47^. Localized molecular orbitals were obtained according to Boys’ method^55^. The geometric and electronic structures were optimized simultaneously; resulting molecular structures are summarized in Supplementary Table 9. Additional reaction steps and corresponding reaction energies are given in Supplementary Figs. 33 and 34.

## Supplementary information

Supplementary Information

Description of Additional Supplementary Files

Supplementary Data 1.cif

Supplementary Data 2.cif

Supplementary Data 3.cif

Supplementary Data 4.cif

Supplementary Data 5.cif

Supplementary Data 6.cif

## Data Availability

All data generated or analyzed during this study are included in this published article and its supplementary information files. The structures of compounds [K(crypt-222)]_3_[η^3^-Bi_3_W(CO)_3_]∙3en∙3tol ([K(crypt-222)]_3_**1**∙3en∙3tol), [K(crypt-222)]_2_[W(CO)_4_(H)_2_]∙en ([K(crypt-222)]_2_**2**∙en), [K(crypt-222)]_3_[µ:η^3^-Bi_3_{W(CO)_3_}_2_]∙en∙tol ([K(crypt-222)]_3_**3**∙en∙tol), [K(crypt-222)]_3_[µ^2^:η^3^-Bi_3_{W(CO)_3_W(CO)_4_}]∙3en∙tol ([K(crypt-222)]_3_**4**∙3en∙tol), and [K(crypt)]_2_[W_2_CO_8_(µ-H_2_)] ∙ 0.5tol ([K(crypt-222)]_2_**5** ∙ 0.5tol) were determined by single-crystal X-ray diffraction. The X-ray crystallographic coordinates for structures reported in this Article have been deposited at the Cambridge Crystallographic Data Centre (CCDC), under deposition numbers CCDC-2239217 ([K(crypt-222)]_3_**1**∙3en∙3tol), CCDC-2239218 ([K(crypt-222)]_2_**2**∙en), CCDC-2239219 ([K(crypt-222)]_3_**3**∙en∙tol), CCDC-2239220 ([K(crypt-222)]_3_**4**∙3en∙tol), and CCDC-2239221 ([K(crypt-222)]_2_**5** ∙ 0.5tol). These data can be obtained free of charge from The Cambridge Crystallographic Data Centre via www.ccdc.cam.ac.uk/data_request/cif. The information is also provided as Supplementary Data 1–5 (CIF format). The Cartesian coordinates of all optimized structures and the respective SCF energies are summarized in Supplementary Data 6 (ASCII format). The files comprise all the necessary data for reproducing the values.

## Code availability

The TURBOMOLE quantum program suite is available from https://www.turbomole.org (retrieved January 27, 2023).
