# Supplementary material for: Insight into the formation of bismuth-tungsten carbonyl clusters
Source: Commun Chem. 2023 Jun 5;6:109. doi: 10.1038/s42004-023-00905-6 (PMC10241888; doi:10.1038/s42004-023-00905-6)
Supplement: Supplementary file 1 — Supplementary Information [file 42004_2023_905_MOESM1_ESM.pdf]

Supplementary Information

Insight into the Formation of Bismuth-Tungsten Carbonyl Clusters

Katrin Beuthert,[1‡] Benjamin Peerless,[1‡] and Stefanie Dehnen*[1]

1 Karlsruhe Institute of Technology (KIT), Institute of Nanotechnology (INT),
P.O. Box, 76021 Karlsruhe, Germany
Corresponding Authors E–mail: stefanie.dehnen@kit.edu
‡ These authors contributed equally

Contents

1. Supplementary Information on the Experiments 2
2. Supplementary Information on Micro X-ray Fluorescence Spectroscopy (*μ*-XFS) Analysis 3
3. Electrospray Ionization Mass Spectrometry (ESI-MS) Investigations 7
3.1 Mass spectrum of the reaction after 24 h 7
4. Supplementary Information on Single Crystal X-ray Diffraction 11
4.1 Crystal Structure of $[K(crypt\text{-}222)]_3[\eta^3\text{-}Bi_3W(CO)_3]\cdot3en\cdot3tol$ ($[K(crypt\text{-}222)]_3$**1**$\cdot3en\cdot3tol$ 12
4.2 Crystal Structure of $[K(crypt\text{-}222)]_2[W(CO)_4(H)_2]\cdot en$ ($[K(crypt\text{-}222)]_2$**2**$\cdot en$) 14
4.3 Crystal Structure of $[K(crypt\text{-}222)]_3[\mu{:}\eta^3\text{-}Bi_3\{W(CO)_3\}_2]\cdot en\cdot tol$ ($[K(crypt\text{-}222)]_3$**3**$\cdot en\cdot tol$) 16
4.4 Crystal Structure of $[K(crypt\text{-}222)]_3[\mu^2{:}\eta^3\text{-}Bi_3\{W(CO)_3W(CO)_4\}]\cdot3en\cdot tol$ ($[K(crypt\text{-}222)]_3$**4**$\cdot3en\cdot tol$) 18
4.5 Crystal Structure of and $[K(crypt)]_2[W_2CO_8(\mu\text{-}H_2)]\cdot0.5tol$ ($[K(crypt\text{-}222)]_2$**5**$\cdot0.5tol$) 20
4.6 Supplementary Crystal Structure 22
4.7 Responses to A- and B-Alerts in the CIF Files of the Crystal Structures 25
5. Infrared Spectroscopy 26
6. Supplementary Information on the Quantum Chemical Investigations 27
6.1 Optimized Molecules, Atoms, and Anions 27
6.2 Calculated Vibrational Spectra 29
7. Supplementary Information on the Reaction Pathways 32
7.1 Supplementary Calculated Reaction Pathways and Corresponding Reaction Energies 32

1. Supplementary Information on the Experiments

Supplementary Figure 1 shows pictures of the reaction solutions and the corresponding crystals of compounds $[K(crypt\text{-}222)]_3[\eta^3\text{-}Bi_3W(CO)_3]\cdot 3en\cdot 3tol$ ($[K(crypt\text{-}222)]_3$**1**$\cdot 3en\cdot 3tol$), $[K(crypt\text{-}222)]_2[W(CO)_4(H)_2]\cdot en$ ($[K(crypt\text{-}222)]_2$**2**$\cdot en$), $[K(crypt\text{-}222)]_3[\mu\text{:}\eta^3\text{-}Bi_3\{W(CO)_3\}_2]\cdot en\cdot tol$ ($[K(crypt\text{-}222)]_3$**3**$\cdot en\cdot tol$), '$[K(crypt\text{-}222)]_5[K\{\mu\text{:}\eta^3\text{-}Bi_3\{W(CO)_3\}_2\}_2]\cdot 2en$', and $[K(crypt)]_2[W_2CO_8(\mu\text{-}H_2)]\cdot 0.5tol$ ($[K(crypt\text{-}222)]_2$**5**$\cdot 0.5tol$).

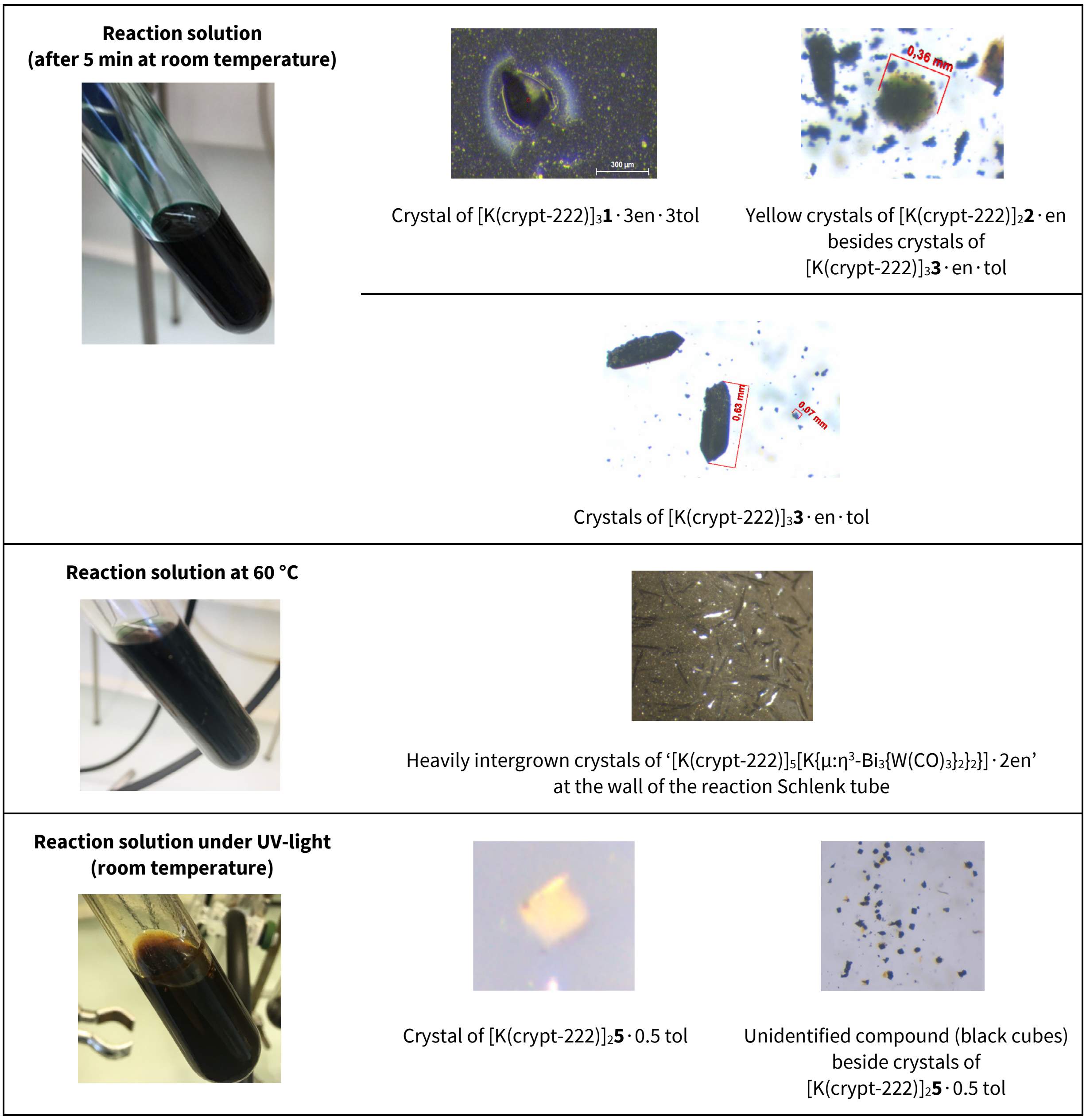

Supplementary Figure 1. Photographs and light-microscopic images of the reaction solutions and the crystals of compounds $[K(crypt\text{-}222)]_3[\eta^3\text{-}Bi_3W(CO)_3]\cdot 3en\cdot 3tol$ ($[K(crypt\text{-}222)]_3 1\cdot 3en\cdot 3tol$), $[K(crypt\text{-}222)]_2[W(CO)_4(H)_2]\cdot en$ ($[K(crypt\text{-}222)]_2 2\cdot en$), $[K(crypt\text{-}222)]_3[\mu\text{:}\eta^3\text{-}Bi_3\{W(CO)_3\}_2]\cdot en\cdot tol$ ($[K(crypt\text{-}222)]_3 3\cdot en\cdot tol$), '$[K(crypt\text{-}222)]_5[K\{\mu\text{:}\eta^3\text{-}Bi_3\{W(CO)_3\}_2\}_2]\cdot 2en$', and $[K(crypt)]_2[W_2CO_8(\mu\text{-}H_2)]\cdot 0.5tol$ ($[K(crypt\text{-}222)]_2 5\cdot 0.5tol$).

2. Supplementary Information on Micro X-ray Fluorescence Spectroscopy (µ-XFS) Analysis

The data of all compounds were collected on crystals from the same reaction the crystal structures were obtained from. Several measurements produced slightly deviating values for the % K. This is observed commonly for these very air-sensitive compounds. We assume that this is due to beginning corrosion on the crystal surface upon air exposure during sample preparation.

Results are summarised in **Supplementary Table 1**. **Supplementary Figures 2 – 5** present the spectra for $[K(crypt\text{-}222)]_3[\eta^3\text{-}Bi_3W(CO)_3]\cdot3en\cdot3tol$ ($[K(crypt\text{-}222)]_3$**1**$\cdot3en\cdot3tol$), $[K(crypt\text{-}222)]_2[W(CO)_4(H)_2]\cdot en$ ($[K(crypt\text{-}222)]_2$**2**$\cdot en$), $[K(crypt\text{-}222)]_3[\mu{:}\eta^3\text{-}Bi_3\{W(CO)_3\}_2]\cdot en\cdot tol$ ($[K(crypt\text{-}222)]_3$**3**$\cdot en\cdot tol$), $[K(crypt\text{-}222)]_3[\mu^2{:}\eta^3\text{-}Bi_3\{W(CO)_3W(CO)_4\}]\cdot3en\cdot tol$ ($[K(crypt\text{-}222)]_3$**4**$\cdot3en\cdot tol$), and $[K(crypt)]_2[W_2CO_8(\mu\text{-}H_2)]\cdot0.5tol$ ($[K(crypt\text{-}222)]_2$**5**$\cdot0.5tol$). The results confirm the values observed by crystallography.

Supplementary Table 1. Summarised results of the µ-XFS analysis of $[K(crypt\text{-}222)]_3[\eta^3\text{-}Bi_3W(CO)_3]\cdot3en\cdot3tol$ ($[K(crypt\text{-}222)]_3$**1**$\cdot3en\cdot3tol$), $[K(crypt\text{-}222)]_2[W(CO)_4(H)_2]\cdot en$ ($[K(crypt\text{-}222)]_2$**2**$\cdot en$), $[K(crypt\text{-}222)]_3[\mu{:}\eta^3\text{-}Bi_3\{W(CO)_3\}_2]\cdot en\cdot tol$ ($[K(crypt\text{-}222)]_3$**3**$\cdot en\cdot tol$), $[K(crypt\text{-}222)]_3[\mu^2{:}\eta^3\text{-}Bi_3\{W(CO)_3W(CO)_4\}]\cdot3en\cdot tol$ ($[K(crypt\text{-}222)]_3$**4**$\cdot3en\cdot tol$), and $[K(crypt)]_2[W_2CO_8(\mu\text{-}H_2)]\cdot0.5tol$ ($[K(crypt\text{-}222)]_2$**5**$\cdot0.5tol$).

Compound	Element-Series	Element wt %	Atom %	Element ratio observed	Element ratio calculated
$[K(crypt\text{-}222)]_3$**1**$\cdot3en\cdot3tol$	K-K	15.79	49.18	4.06	3
	W-L	21.91	14.51	1.19	1
	Bi-L	62.30	36.31	3.00	3
	Total	100	100		
$[K(crypt\text{-}222)]_2$**2**$\cdot en$	K-K	38.89	75.23	3.42	2
	W-L	53.50	22.01	1.00	1
	Bi-L	7.62	2.76	0.13	0
	Total	100	100		
$[K(crypt\text{-}222)]_3$**3**$\cdot en\cdot tol$	K-K	10.67	37.79	3.07	3
	W-L	33.62	25.31	2.06	2
	Bi-L	55.71	36.90	3.00	3
	Total	100	100		
$[K(crypt\text{-}222)]_3$**4**$\cdot3en\cdot tol$	K-K	14.59	46.79	3.81	3
	W-L	24.03	16.39	2.25	2
	Bi-L	61.38	36.82	3.00	3
	Total	100	100		
$[K(crypt\text{-}222)]_2$**5**$\cdot0.5tol$	K-K	20.63	55.07	2.50	2
	W-L	77.60	44.05	2.00	2
	Bi-L	1.76	0.88	0.04	0
	Total	100	100		

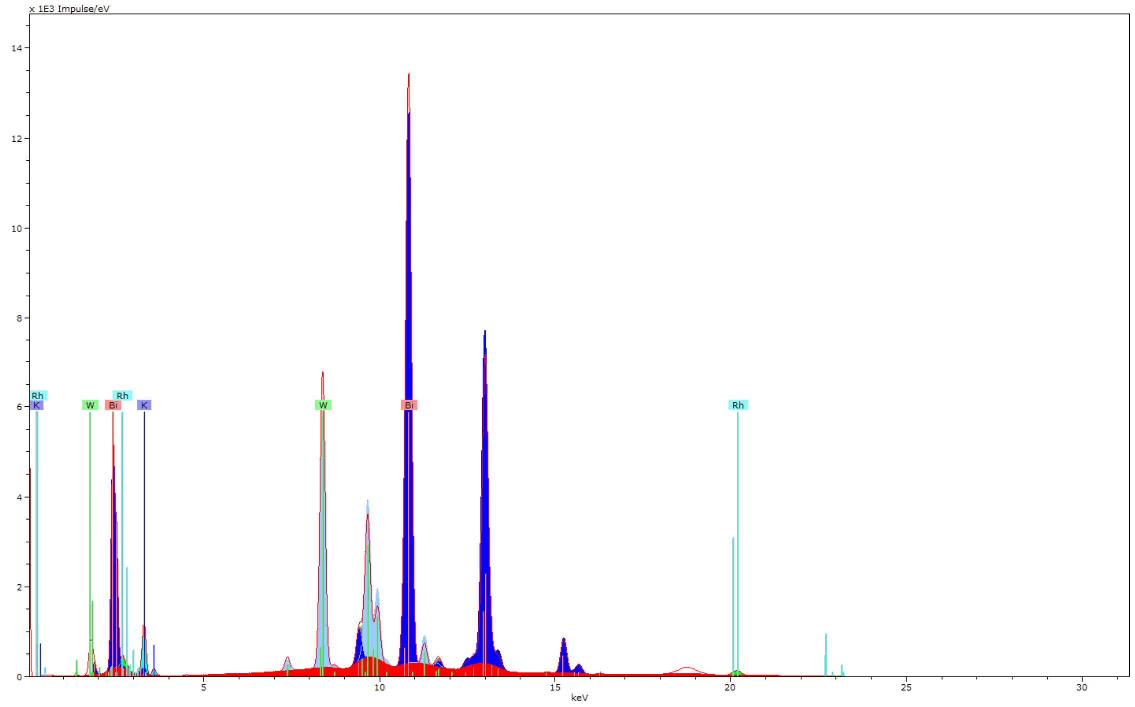

Supplementary Figure 2. μ-XFS spectrum of [K(crypt-222)]$_3$[η^3-Bi$_3$W(CO)$_3$]·3en·3tol ([K(crypt-222)]$_3$1·3en·3tol. Values (% found) for the elemental composition of K:W:Bi was detected as follows: K: 49.18, W: 14.51, Bi: 36.31, corresponding to atomic ratios of: 3:1:3.

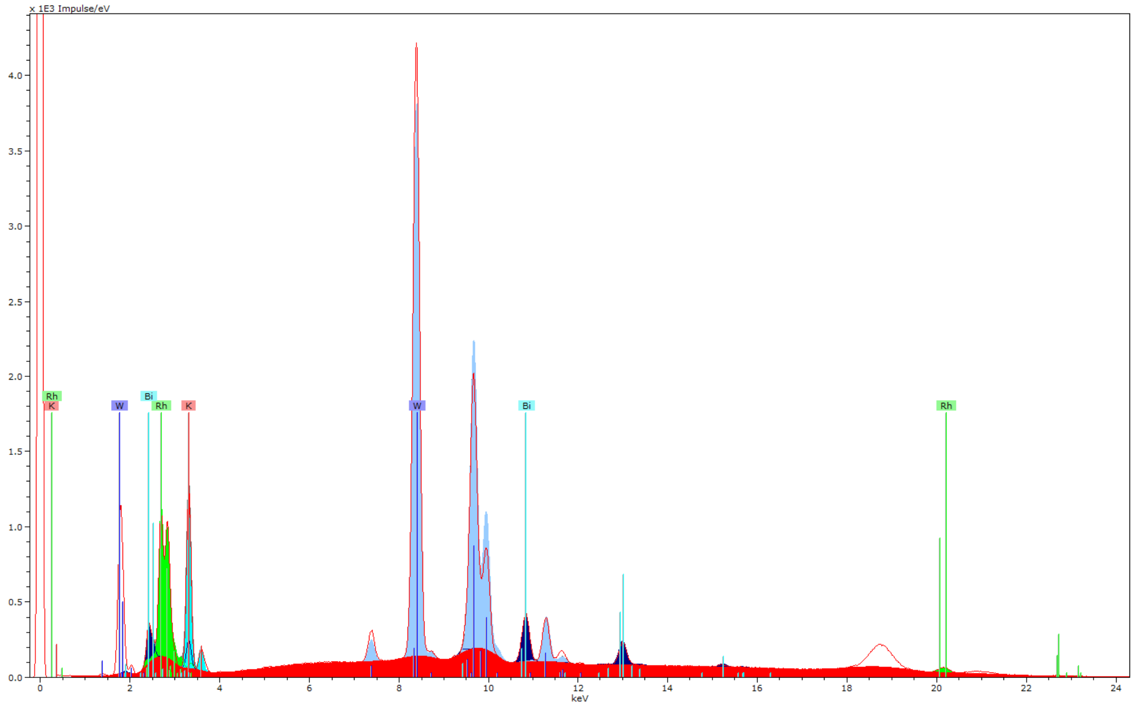

Supplementary Figure 3. μ-XRF spectrum of [K(crypt-222)]$_2$[W(CO)$_4$(H)$_2$]·en ([K(crypt-222)]$_2$2·en). Values (% found) for the elemental composition of K:W:Bi was detected as follows: K: 75.23, W: 22.01, Bi: 2.76, corresponding to atomic ratios of: 2:1:0.

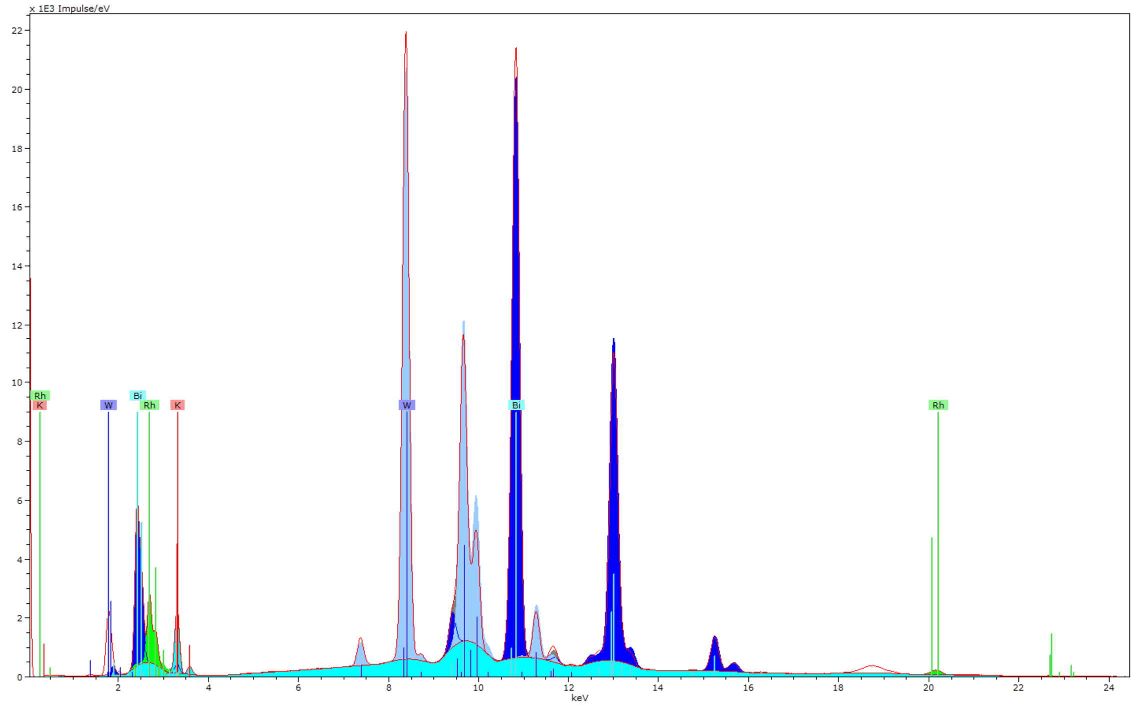

Supplementary Figure 4. μ-XRF spectrum of [K(crypt-222)]$_3$[μ:η^3-Bi$_3${W(CO)$_3$}$_2$]·en·tol ([K(crypt-222)]$_3$3·en·tol). Values (% found) for the elemental composition of K:W:Bi was detected as follows: K: 37.79, W: 25.31, Bi: 36.90, corresponding to atomic ratios of: 3:2:3.

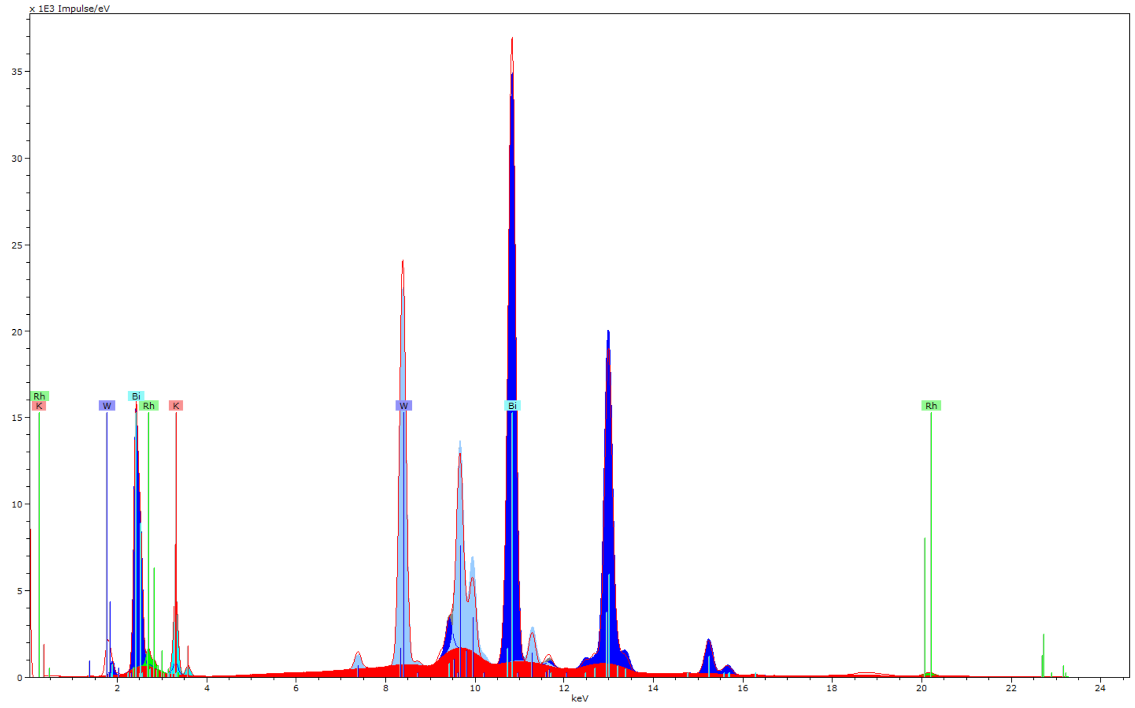

Supplementary Figure 5. μ-XRF spectrum of [K(crypt-222)]$_3$[μ^2:η^3-Bi$_3${W(CO)$_3$W(CO)$_4$}]·3en·tol ([K(crypt-222)]$_3$4·3en·tol). Values (% found) for the elemental composition of K:W:Bi was detected as follows: K: 46.79, W: 16.39, Bi: 36.82, corresponding to atomic ratios of: 3:2:3.

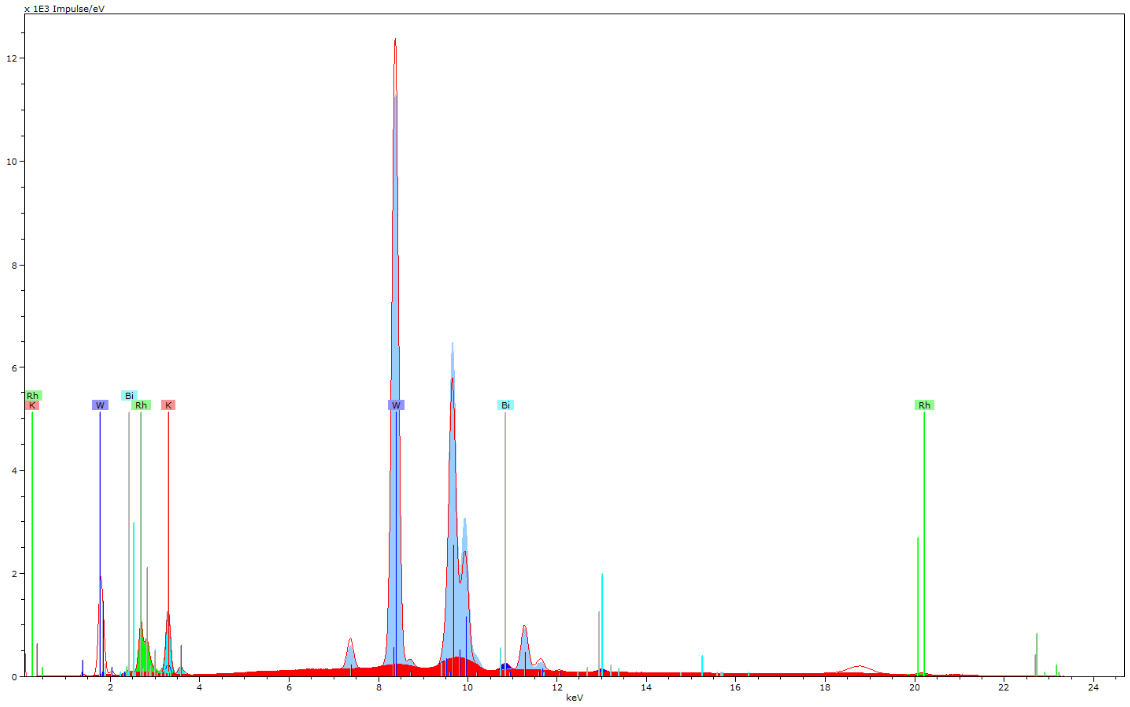

Supplementary Figure 6. μ-XRF spectrum of [K(crypt)]$_2$[W_2CO_8(μ-H_2)]·0.5tol ([K(crypt-222)]$_2$5·0.5tol). Values (% found) for the elemental composition of K:W:Bi was detected as follows: K: 55.07, W: 44.05, Bi: 0.88, corresponding to atomic ratios of: 2:2:0.

3. Electrospray Ionization Mass Spectrometry (ESI-MS) Investigations

3.1 Mass spectrum of the reaction after 24 h

Supplementary Figure 7 shows a mass spectrum recorded 24 hours after the start of the reaction upon injection of the filtered reaction solution into the spectrometer. **Supplementary Table 2** gives an overview of the identified signals. The corresponding assigned high-resolution mass peaks are shown in **Supplementary Figures 8 – 12**. Several attempts to measure mass spectra of the compounds and the reaction solutions failed due to clogging of the PTFE tube. We assume that the clusters are fragmented directly at the beginning of the measurement, which is why we can only see the smaller fragments in the mass spectrum.

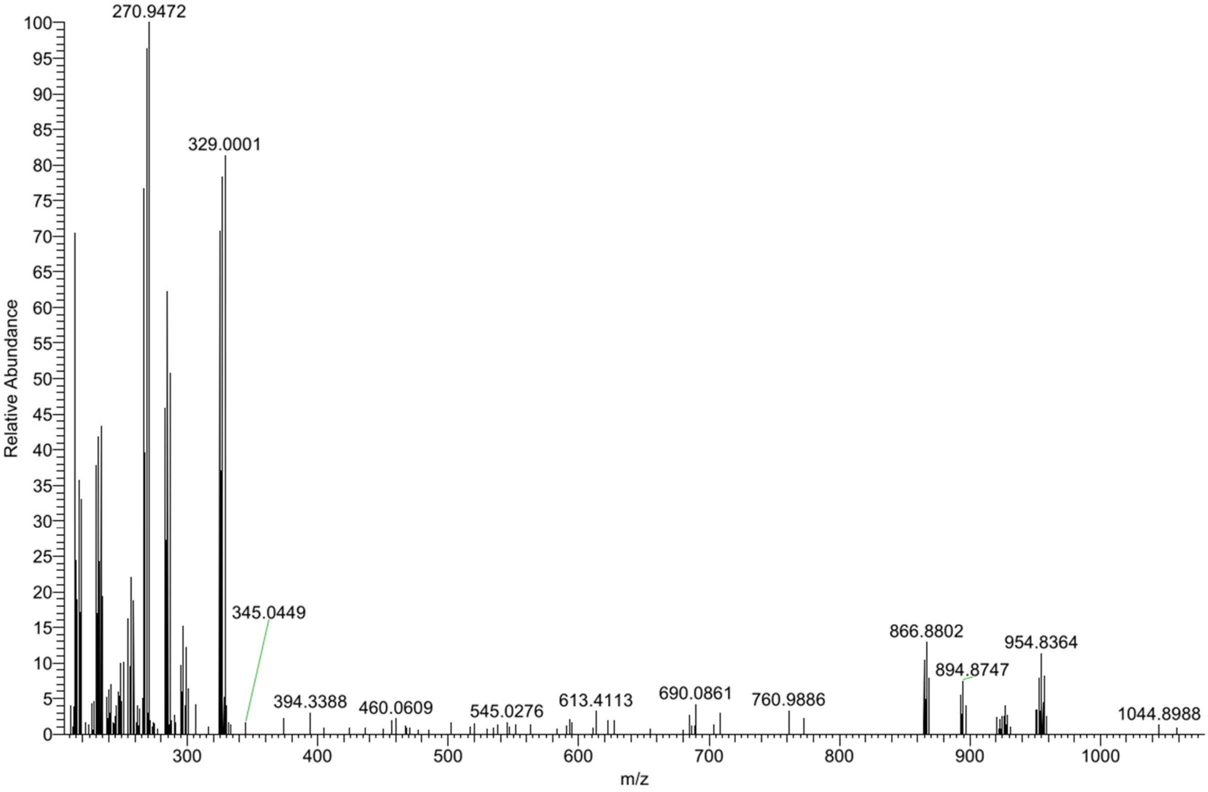

Supplementary Figure 7. Overview of ESI(−) mass spectrum recorded immediately upon injection of a filtered reaction solution after 24 h at RT in en.

Supplementary Table 2. Signals in the ESI mass spectrum of the reaction after 24 h.

main signal in isotopic pattern (m/z)	elemental composition
268.9438	$(W_1C_3O_3H)^-$
296.9861	$(W_1C_4O_4H)^-$
866.8802	$(W_1Bi_3C_2O_2)^-$
894.8747	$(W_1Bi_3C_3O_3)^-$
924.8405	$(W_1Bi_3C_4O_4)^-$

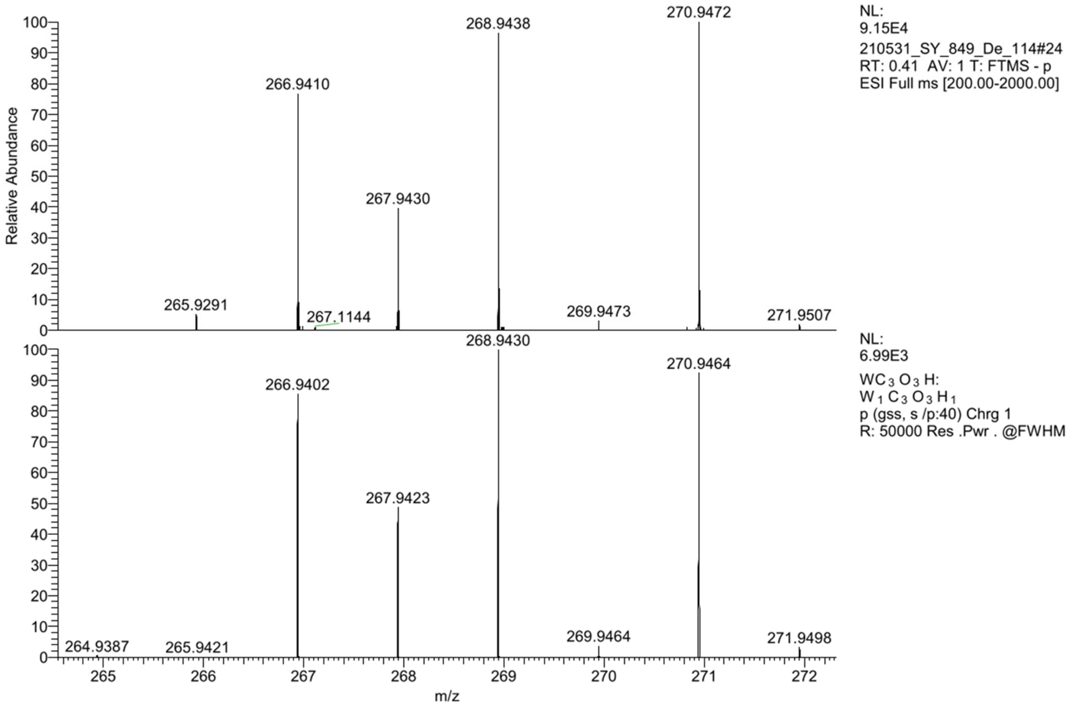

Supplementary Figure 8. High-resolution ESI mass spectrum in negative ion mode recorded immediately upon injection of the reaction solution after 24 h in en, indicating the existence of $(W_1C_3O_3H_1)^-$. Topmost: measured, below: simulated.

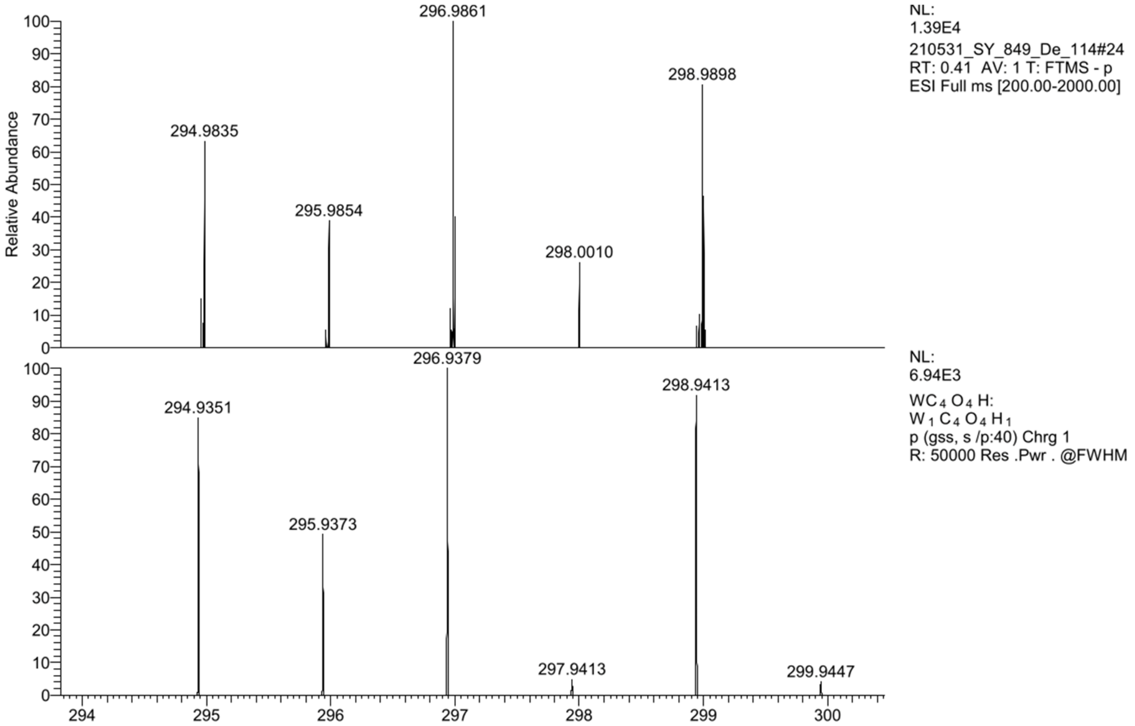

Supplementary Figure 9. High-resolution ESI mass spectrum in negative ion mode recorded immediately upon injection of the reaction solution after 24 h in en, indicating the existence of $(W_1C_4O_4H_1)^-$. Topmost: measured, below: simulated.

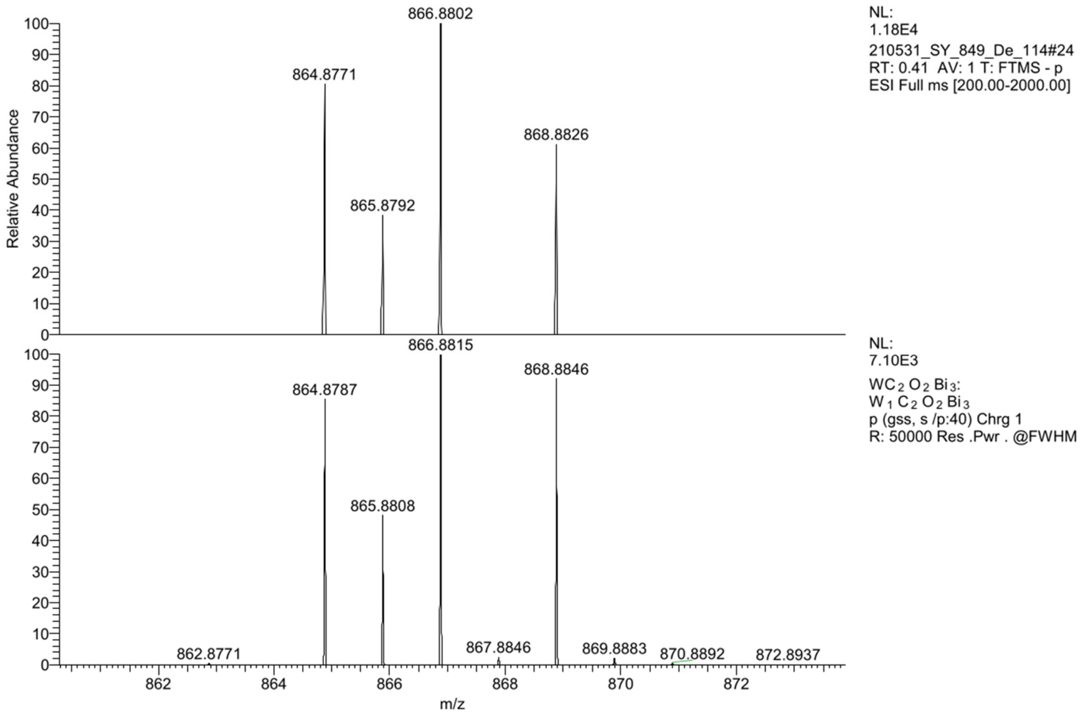

Supplementary Figure 10. High-resolution ESI mass spectrum in negative ion mode recorded immediately upon injection of the reaction solution after 24 h in en, indicating the existence of $(W_1Bi_3C_2O_2)^-$. Topmost: measured, below: simulated.

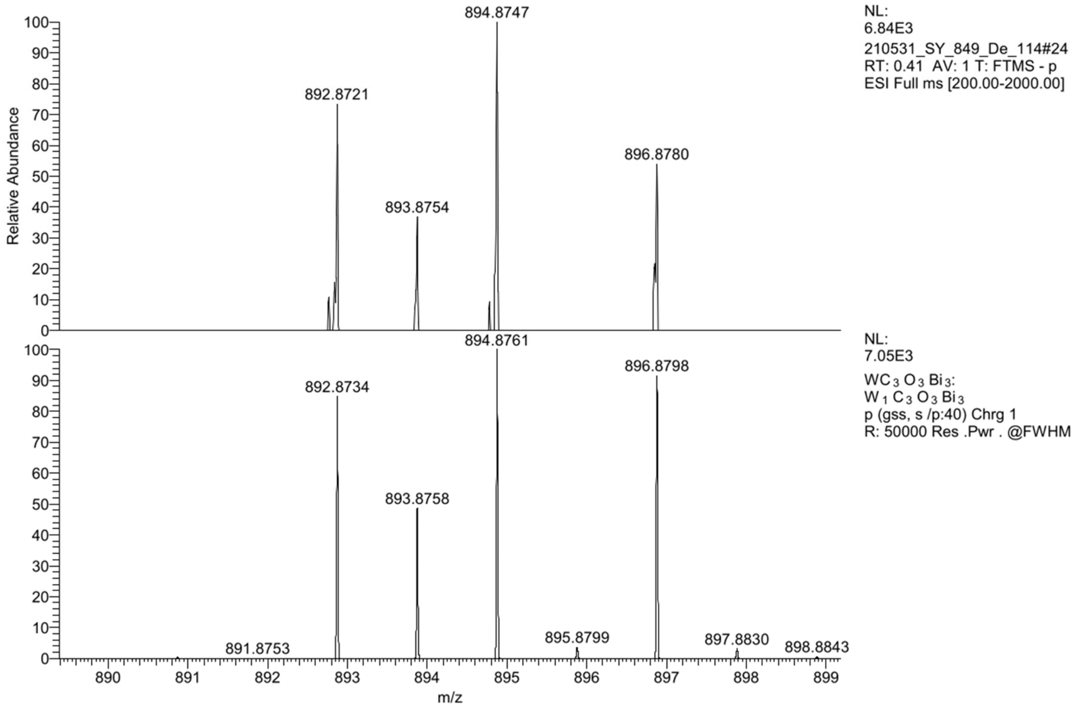

Supplementary Figure 11. High-resolution ESI mass spectrum in negative ion mode recorded immediately upon injection of the reaction solution after 24 h in en, indicating the existence of $(W_1Bi_3C_3O_3)^-$. Topmost: measured, below: simulated.

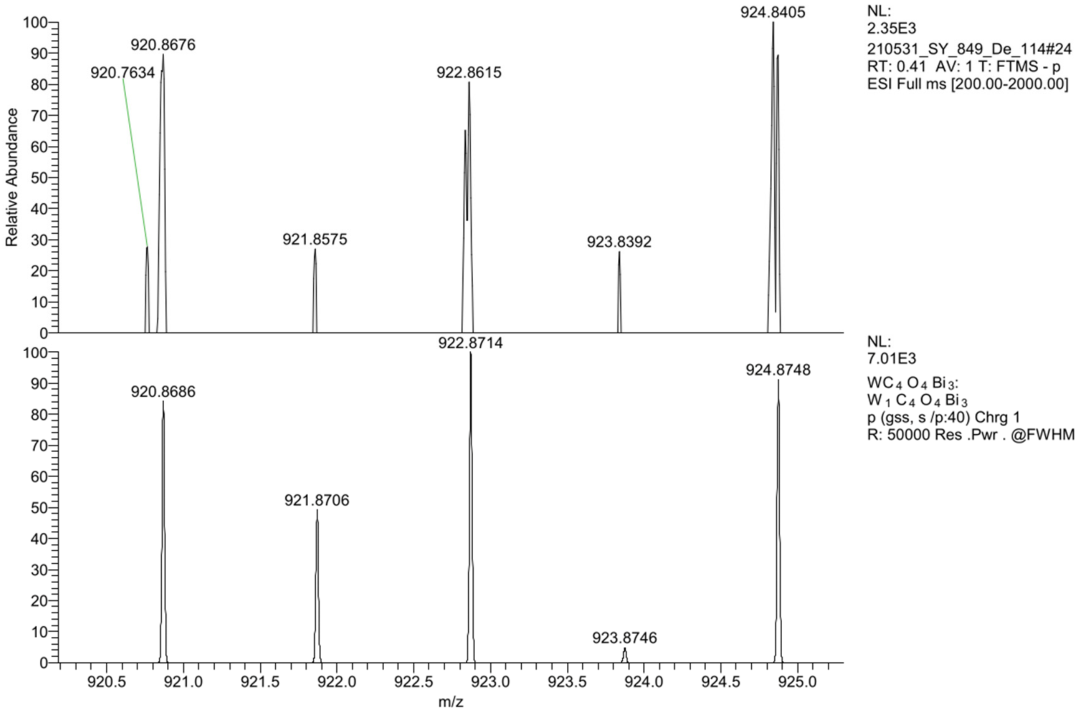

Supplementary Figure 12. High-resolution ESI mass spectrum in negative ion mode recorded immediately upon injection of the reaction solution after 24 h in en, indicating the existence of $(W_1Bi_3C_4O_4)^-$. Topmost: measured, below: simulated.

4. Supplementary Information on Single Crystal X-ray Diffraction

Crystallographic data for the structures reported in this paper have been deposited with the Cambridge Crystallographic Data Centre as supplementary publications:

- CCDC- 2239217 ([K(crypt-222)]**1**·3en·3tol)
- CCDC- 2239218 ([K(crypt-222)]**2**·en)
- CCDC- 2239219 ([K(crypt-222)]**3**·en·tol)
- CCDC- 2239220 ([K(crypt-222)]**4**·3en·tol)
- CCDC- 2239221 ([K(crypt-222)]**5**·0.5tol)

The crystal data and experimental parameters of the structure determinations are collected in the **Supplementary Tables 3 – 8**. Supplementary structural figures are provided in **Supplementary Figure 13 – 26**.

4.1 Crystal Structure of $[K(crypt\text{-}222)]_3[\eta^3\text{-}Bi_3W(CO)_3]\cdot 3en\cdot 3tol$ ($[K(crypt\text{-}222)]_3$**1**$\cdot 3en\cdot 3tol$)

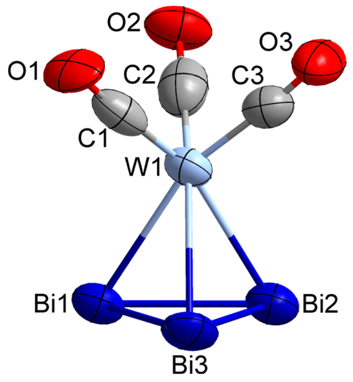

Supplementary Figure 13. Molecular structure of the $[W(CO)_3Bi_3]^{3-}$ anion. Illustration without the disorder of Bi1, Bi2 and Bi3. W, Bi, C and O are shown with 50 % probability.

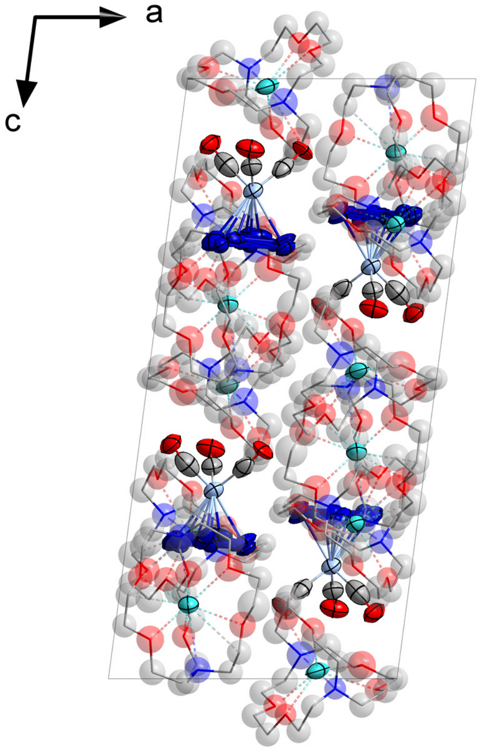

Supplementary Figure 14. Excerpt of the crystal structure of 1 viewed along the *b* axis with W = light blue, Bi = dark blue, K = turquoise, C = grey, O = red, N = blue. H atoms are omitted for clarity. W, Bi, and K are shown with 50 % probability. C, N and O atoms are shown in the ball-and-stick model.

A solvent mask was calculated and 574 electrons were found in a volume of 1966 Å^3 in 2 voids per unit cell. This is consistent with the presence of 3 $[C_2N_2H_8]$ (en), 1 $[C_7H_8]$ (tol) per asymmetric unit which account for 608 electrons per unit cell.

Supplementary Table 3. Crystallographic data and refinement results of [K(crypt-222)]$_3$1 · 3en · 3tol.

Compound	[K(crypt-222)]$_3$1 · 3en · 3tol
Empirical Formula	$C_{57}H_{108}Bi_3K_3N_6O_{21}W$
Formula weight / g mol^{-1}	2141.58
Crystal colour and shape	Black block
Crystal size / mm	0.08 x 0.06 x 0.03
Crystal system	Monoclinic
Space group type	$P2_1/c$
a / Å	13.1074(4)
b / Å	25.2633(6)
c / Å	27.5112(8)
α / °	90
β / °	97.498(2)
γ / °	90
V / Å^3	9032.1(4)
Z	4
Measurement Temperature / K	100
ρ_{calcd} / g cm^{-3}	1.575
$\mu_{(Cu\ K\alpha)}$ / mm^{-1}	7.293
Absorption correction type	Multi-scan
Min./max. transmission	1.000 / 1.000
2θ range / deg	2.98 – 46.31
No. of measured reflections	31795
R(int)	0.047
Independent Reflections	8579
No. of parameters	475
Restraints	88
R_1 / wR_2 (I>2σ (I))	0.0991 / 0.2821
R_1 / wR_2 (all data)	0.1206 / 0.2980
S (all data)	1.081
Max. peak / hole / e$^-$ Å^{-3}	3.200 / −1.900
CCDC number	2239217

4.2 Crystal Structure of $[K(crypt\text{-}222)]_2[W(CO)_4(H)_2]\cdot en$ ($[K(crypt\text{-}222)]_2\mathbf{2}\cdot en$)

Because of the high electron density around the W atom it was not possible to refine the H atoms. The max. electron density peak is 1.811 Å away from the W atom.

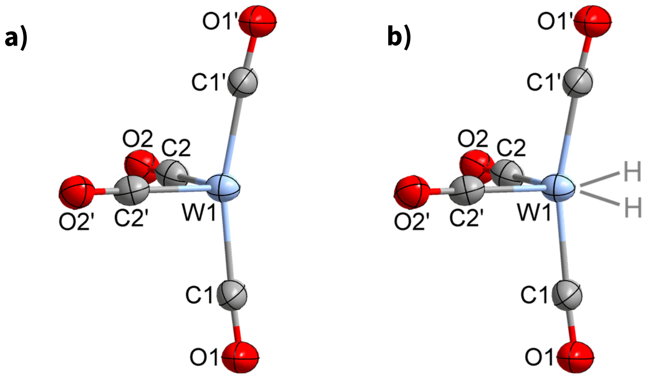

Supplementary Figure 15. Molecular structure of the $[W(CO)_4]^{2-}$ anion. W, C and O atoms are shown with 50 % probability. a) Without H atoms at the W atom; b) with H atoms sketched at the W atom.

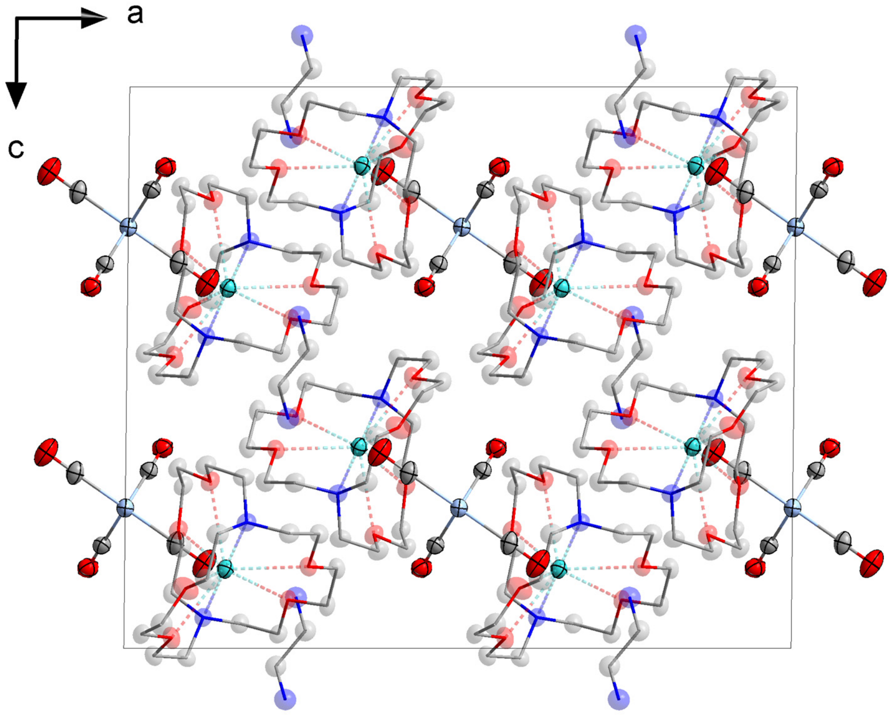

Supplementary Figure 16. Excerpt of the crystal structure of $[K(crypt\text{-}222)]_2\mathbf{2}\cdot en$ viewed along the *b* axis with W = light blue, K = turquoise, C = grey, O = red, N = blue. H atoms are omitted for clarity. K, W, C, N and O atoms are shown with 50 % probability.

Supplementary Table S4. Crystallographic data and refinement results of [K(crypt-222)]₂2·en.

Compound	[K(crypt-222)]$_2$2·en
Empirical Formula	$C_{42}H_{80}K_2N_6O_{16}W$
Formula weight / g mol^{-1}	1187.17
Crystal colour and shape	Yellow plate
Crystal size / mm	0.04 x 0.035 x 0.01
Crystal system	Monoclinic
Space group type	*C*2/*c*
a / Å	21.5280(2)
b / Å	13.73490(10)
c / Å	18.1264(2)
α / °	90
β / °	90.6190(10)
γ / °	90
V / Å^3	5359.39(9)
Z	4
Measurement Temperature / K	100
ρ_{calcd} / g cm^{-3}	1.471
$\mu_{(Cu\ K\alpha)}$ / mm^{-1}	5.958
Absorption correction type	Multi-scan
Min./max. transmission	0.1049 / 0.1586
2θ range / deg	3.818 – 76.378
No. of measured reflections	182791
R(int)	0.0382
Independent Reflections	120165
No. of parameters	316
Restraints	0
R_1 / wR_2 (I>2σ (I))	0.0462 / 0.1250
R_1 / wR_2 (all data)	0.0472 / 0.1262
S (all data)	1.048
Max. peak / hole / e$^-$ Å^{-3}	1.500 / −2.330
CCDC number	2239218

4.3 Crystal Structure of $[K(crypt\text{-}222)]_3[\mu:\eta^3\text{-}Bi_3\{W(CO)_3\}_2]\cdot en\cdot tol$ ($[K(crypt\text{-}222)]_3$**3**$\cdot en\cdot tol$)

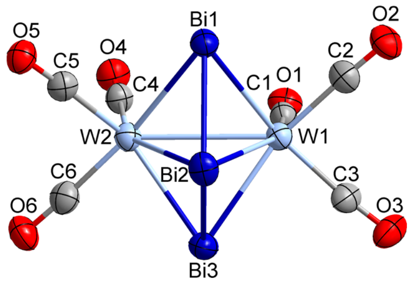

Supplementary Figure 17. Molecular structure of the $[W(CO)_3Bi_3W(CO)_3]^{3-}$ anion. Bi, W, C and O atoms are shown with 50 % probability.

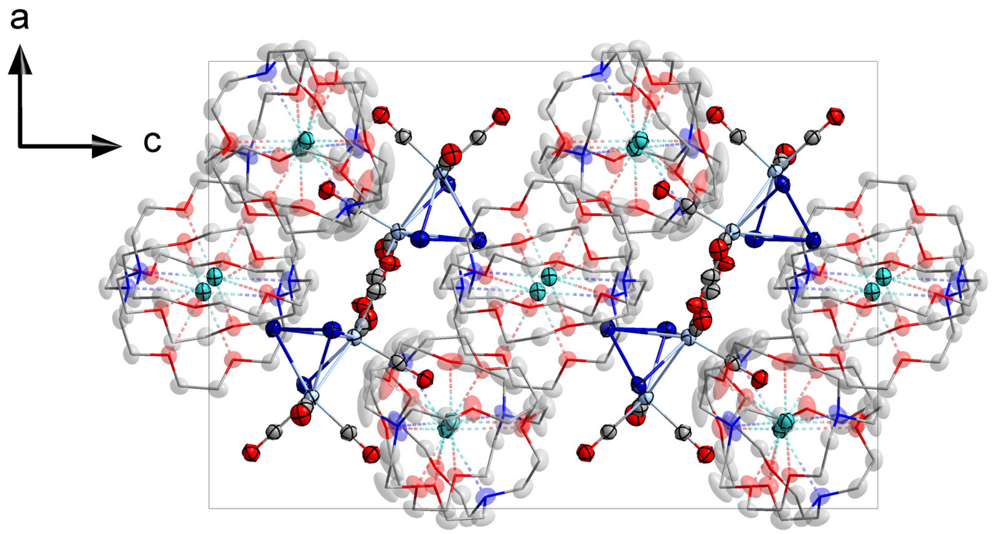

Supplementary Figure 18. Excerpt of the crystal structure of $[K(crypt\text{-}222)]_3$3$\cdot$en$\cdot$tol viewed along the *b* axis with W = light blue, Bi = dark blue, K = turquoise, C = grey, O = red, N = blue. H atoms are omitted for clarity. Bi, W, K, C, N and O atoms are shown with 50 % probability.

A solvent mask was calculated and 360 electrons were found in a volume of 1692 Å^3 in 1 void per unit cell. This is consistent with the presence of 1 $[C_2N_2H_8]$ (en), 1 $[C_7H_8]$ (tol) per asymmetric unit which account for 328 electrons per unit cell.

Supplementary Table 5. Crystallographic data and refinement results of [K(crypt-222)]$_3$3·en·tol.

Compound	[K(crypt-222)]$_3$3·en·tol
Empirical Formula	$C_{60}H_{106}Bi_3K_3N_6O_{24}W_2$
Formula weight / g mol^{-1}	2407.44
Crystal colour and shape	Dark red stick
Crystal size / mm	0.1 x 0.035 x 0.03
Crystal system	Monoclinic
Space group type	$P2_1/c$
a / Å	16.7613(3)
b / Å	21.8199(2)
c / Å	25.1123(4)
α / °	90
β / °	90.0370(10)
γ / °	90
V / Å^3	9184.3(2)
Z	4
Measurement Temperature / K	100
ρ_{calcd} / g cm^{-3}	1.741
$\mu_{(Cu K\alpha)}$ / mm^{-1}	8.42
Absorption correction type	Multi-scan
Min./max. transmission	26.821
2θ range / deg	1.215
No. of measured reflections	72113
R(int)	0.0442
Independent Reflections	15481
No. of parameters	883
Restraints	0
R_1 / wR_2 (I>2σ (I))	0.0359 / 0.0906
R_1 / wR_2 (all data)	0.0461 / 0.0940
S (all data)	0.978
Max. peak / hole / e$^-$ Å^{-3}	2.380 / −0.700
CCDC number	2239219

4.4 Crystal Structure of [K(crypt-222)]$_3$[μ^2:η^3-Bi$_3${W(CO)$_3$W(CO)$_4$}]·3en·tol ([K(crypt-222)]$_3$4·3en·tol)

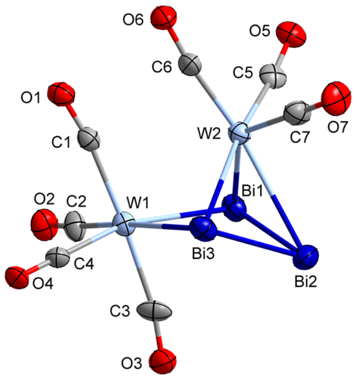

Supplementary Figure 19. Molecular structure of the [W(CO)$_3$Bi$_3$W(CO)$_4$]$^{3-}$ anion. Bi, W, C and O atoms are shown with 50 % probability.

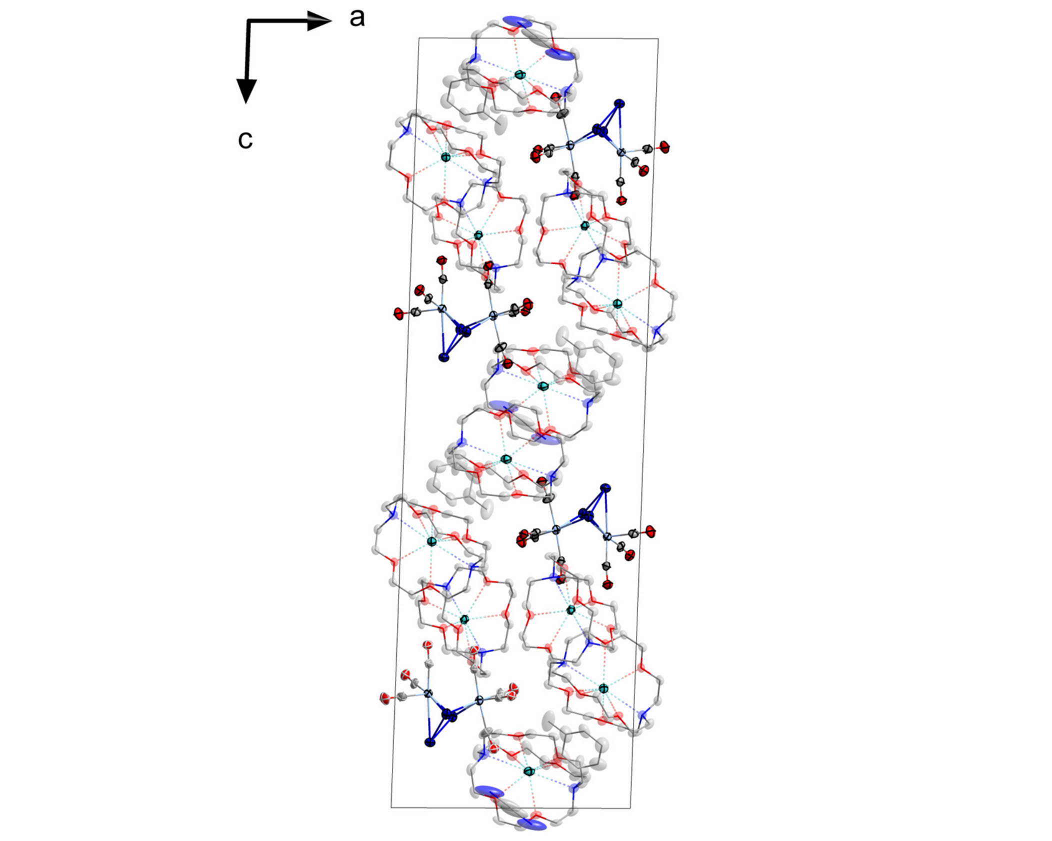

Supplementary Figure 20. Excerpt of the crystal structure of [K(crypt-222)]$_3$4·3en·tol viewed along the *b* axis with W = light blue, Bi = dark blue, K = turquoise, C = grey, O = red, N = blue. H atoms are omitted for clarity. Bi, W, C; N and O atoms are shown with 50 % probability.

A solvent mask was calculated and 262 electrons were found in a volume of 1078 Å^3 in 3 voids per unit cell. This is consistent with the presence of 2 [$C_2N_2H_8$] (en) per asymmetric unit which account for 272 electrons per unit cell.

Supplementary Table S6. Crystallographic data and refinement results of [K(crypt-222)]$_3$4·3en·tol.

Compound	[K(crypt-222)]$_3$4·3en·tol
Empirical Formula	$C_{69}H_{119}Bi_3K_3N_7O_{25}W_2$
Formula weight / g mol^{-1}	2558.64
Crystal colour and shape	Black block
Crystal size / mm	0.18 x 0.16 x 0.12
Crystal system	monoclinic
Space group type	$P2_1/c$
a / Å	15.0896(2)
b / Å	12.8320(2)
c / Å	48.5864(9)
α / °	90
β / °	91.9030(10)
γ / °	90
V / Å^3	9402.6(3)
Z	4
Measurement Temperature / K	100
ρ_{calcd} / g cm^{-3}	1.807
$\mu_{(Cu\ K\alpha)}$ / mm^{-1}	8.235
Absorption correction type	Multi-scan
Min./max. transmission	1.000 / 1.000
2θ range / deg	3.0 – 60.0
No. of measured reflections	107190
R(int)	0.0972
Independent Reflections	19780
No. of parameters	983
Restraints	0
R_1 / wR_2 (I>2σ (I))	0.0626 / 0.1786
R_1 / wR_2 (all data)	0.0918 / 0.1697
S (all data)	1.045
Max. peak / hole / e$^-$ Å^{-3}	2.700 / −2.400
CCDC number	2239220

4.5 Crystal Structure of and $[K(crypt)]_2[W_2CO_8(\mu\text{-}H_2)]\cdot0.5tol$ ($[K(crypt\text{-}222)]_2$5·0.5tol)

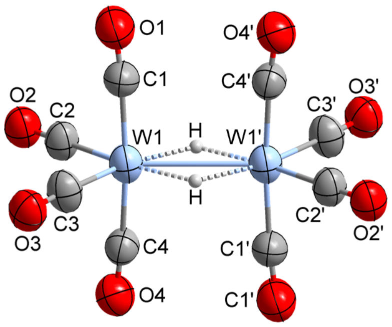

Supplementary Figure 21. Molecular structure of the $[W_2CO_8(\mu\text{-}H_2)]^{2-}$ anion. W, C and O atoms are shown with 50 % probability.

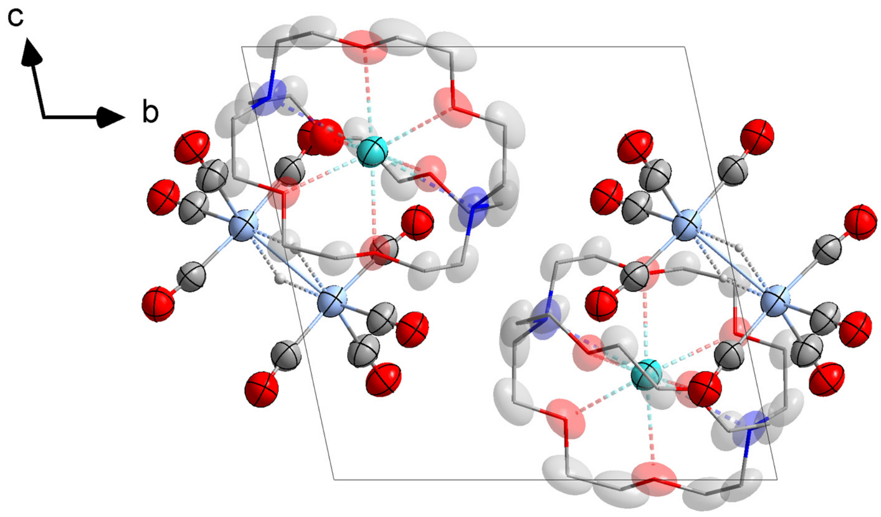

Supplementary Figure 22. Excerpt of the crystal structure of $[K(crypt\text{-}222)]_2$5·0.5 tol viewed along the *a* axis with W = light blue, K = turquoise, C = grey, O = red, N = blue. H atoms of [K(crypt-222)] are omitted for clarity. W, K, C, N and O atoms are shown with 50 % probability.

A solvent mask was calculated and 56 electrons were found in a volume of 240 Å^3 in 1 void per unit cell. This is consistent with the presence of 0.5 $[C_7H_8]$ (tol) per asymmetric unit which account for 50 electrons per unit cell.

Supplementary Table 7. Crystallographic data and refinement results of [K(crypt-222)]$_2$5·0.5 tol.

Compound	[K(crypt-222)]$_2$5·0.5 tol
Empirical Formula	$C_{44}H_{74}K_2N_4O_{20}W_2$
Formula weight / g mol^{-1}	1424.97
Crystal colour and shape	Pale yellow platelet
Crystal size / mm^3	0.15 x 0.08 x 0.02
Crystal system	triclinic
Space group type	$P\bar{1}$
a / Å	12.2519(3)
b / Å	12.8044(3)
c / Å	12.7458(3)
α / °	87.893(2)
β / °	63.398(2)
γ / °	63.057(2)
V / Å^3	1558.80(7)
Z	1
Measurement Temperature / K	100
ρ_{calcd} / g cm^{-3}	1.518
$\mu_{(Cu\ K\alpha)}$ / mm^{-1}	8.479
Absorption correction type	Multi scan
Min./max. transmission	0.011 / 0.022
2θ range / deg	3.960 – 76.282
No. of measured reflections	38344
R(int)	0.0628
Independent Reflections	6375
No. of parameters	330
Restraints	0
R_1 / wR_2 (I>2σ (I))	0.0666 / 0.1963
R_1 / wR_2 (all data)	0.0736 / 0.1890
S (all data)	1.155
Max. peak / hole / e$^-$ Å^{-3}	1.150 / −1.620
CCDC number	2239221

4.6 Supplementary Crystal Structure

By crystallization of the remaining sample after the mass measurement after 2 h reaction time a big octahedral black crystal could be obtained. Because of the high cell volume (approx. 187 000 Å^3), merohedral twinning and a cubic space group, the structure could only be determined but not fully refined. Additionally, a solvent mask was calculated and 14033 electrons were found in a volume of 54008 in 2 voids per unit cell. This is consistent with the presence of 0.75 [$K_1C_{18}N_2H_{36}O_6$] ([K(crypt-222)] disordered due to a special position), 1.5 [$C_2N_2H_8$] (en) per asymmetric unit which account for 14064 electrons per unit cell. Since the co-crystallization of the three anionic units in the structure support the findings reported in this work, the structure is nevertheless shown but not discussed in detail. **Supplementary Figure 23** shows the three anionic units in the crystal structure. **Supplementary Figures 24 – 26** show the unit cell of the crystal structure from different viewing directions.

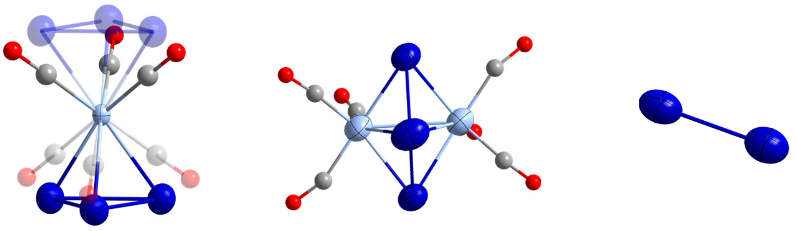

a) c) d)

Supplementary Figure 23. Molecular structures of the three anionic units with Bi = dark blue, W = light blue, C = grey and O = red. Bi and W atoms are shown with 50 % probability. C and O atoms are shown in the balls-and-sticks model.

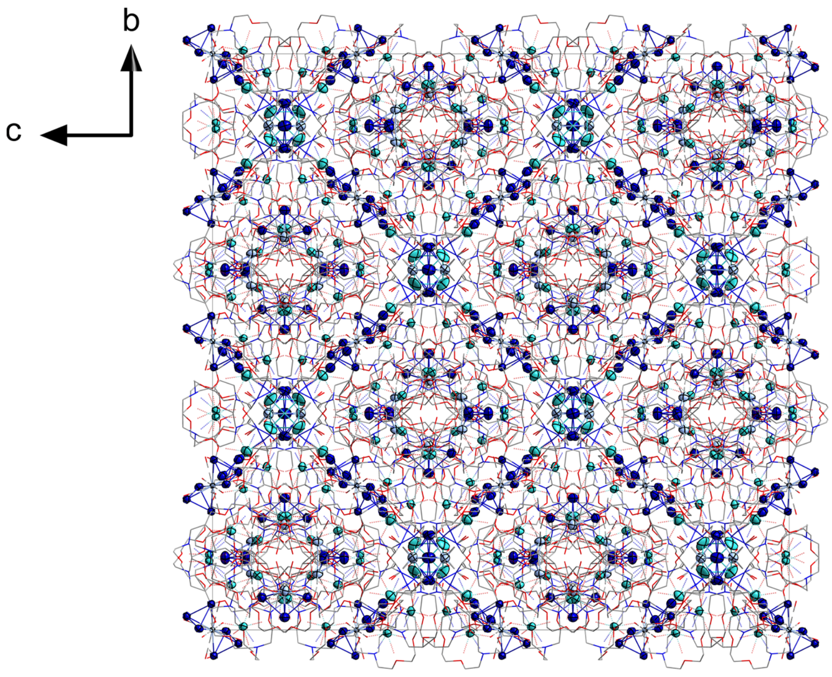

Supplementary Figure 24. Excerpt of the crystal structure viewed along the *a* axis with Bi = dark blue, W = light blue, C = grey and O = red. Bi, W and K atoms are shown with 50 % probability. C, O and N atoms are shown in the balls-and-sticks model. H atoms are omitted for clarity.

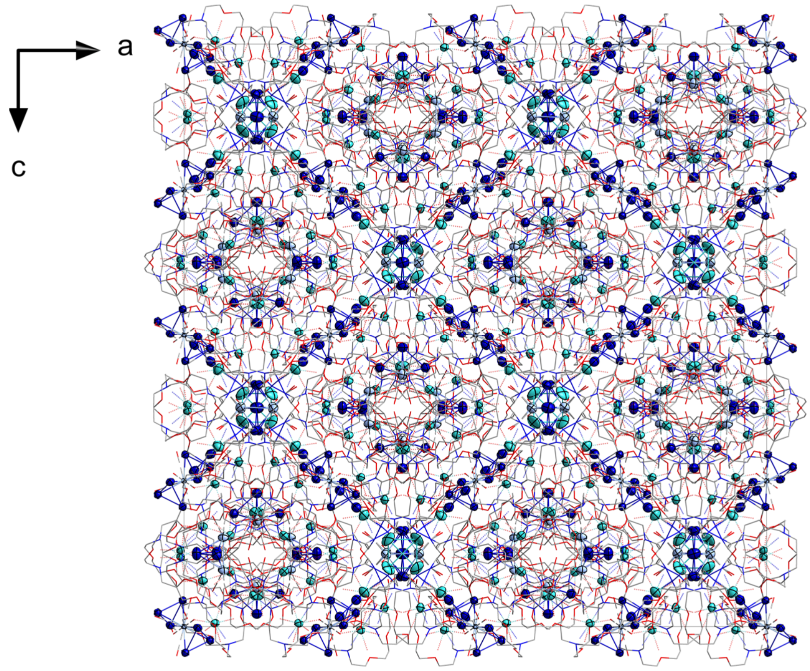

Supplementary Figure 25. Excerpt of the crystal structure viewed along the *b* axis with Bi = dark blue, W = light blue, C = grey and O = red. Bi, W and K atoms are shown with 50 % probability. C, O and N atoms are shown in the balls-and-sticks model. H atoms are omitted for clarity.

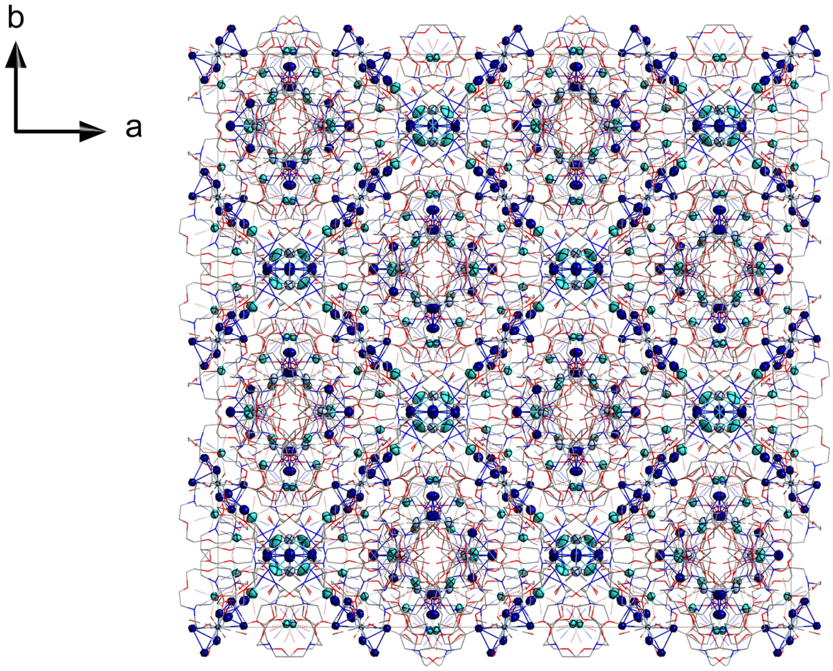

Supplementary Figure 26. Excerpt of the crystal structure viewed along the *c* axis with Bi = dark blue, W = light blue, C = grey and O = red. Bi, W and K atoms are shown with 50 % probability. C, O and N atoms are shown in the balls-and-sticks model. H atoms are omitted for clarity.

Supplementary Table 8. Crystallographic data and refinement results of the supplementary crystal structure.

Compound	Supplementary Crystal Structure
Empirical Formula	$C_{67.5}H_{114}Bi_4K_{3.6}N_8O_{27}W_{1.6}$
Formula weight / g mol^{-1}	2631.18
Crystal colour and shape	Black octahedra
Crystal size / mm^3	0.09 x 0.09 x 0.07
Crystal system	cubic
Space group type	$Fd\bar{3}$
a / Å	57.280(7)
b / Å	57.280(7)
c / Å	57.280(7)
α / °	90
β / °	90
γ / °	90
V / Å^3	187 936(38)
Z	64
Measurement Temperature / K	100
ρ_{calc} / g cm^{-3}	1.553
$\mu_{(Cu\ K\alpha)}$ / mm^{-1}	16.02
Absorption correction type	Multi-scan
Min./max. transmission	0.8870 /0.9291
2θ range / deg	5.116 – 159.108
No. of measured reflections	664529
R(int)	0.1398
Independent Reflections	17002
No. of parameters	340
Restraints	6
R_1 / wR_2 (I>2σ (I))	0.1149 / 0.0348
R_1 / wR_2 (all data)	0.1450 / 0.0350
S (all data)	1.1700
Max. peak / hole / e$^-$ Å^{-3}	25.4 / −1.85

4.7 Responses to A- and B-Alerts in the CIF Files of the Crystal Structures

Compound $[K(crypt\text{-}222)]_3[\eta^3\text{-}Bi_3W(CO)_3]\cdot 3en\cdot 3tol$ ($[K(crypt\text{-}222)]_3$1$\cdot$3en$\cdot$3tol)

PROBLEM: The value of sine(theta_max)/wavelength is less than 0.550
RESPONSE: This is due to limited crystal quality.

PROBLEM: Isotropic non-H Atoms in Main Residue(s) .... 78 Report
RESPONSE: Due to disorder, some of the organic groups needed to be refined using isotropic displacement parameters.

PROBLEM: Isolated Metal Atom found in Structure (Unusual) Bi3 Check
RESPONSE: This is a software error that does not account for the given Bi-Bi and Bi-W bond lengths.

PROBLEM: Single Bonded Metal Atom in Structure (Unusual) Bi1 Check
RESPONSE: This is a software error that does not account for the given Bi-Bi and Bi-W bond lengths.

PROBLEM: Short Intra H...H Contact H14B ..H16B . 1.75 Ang.
RESPONSE: This is due to disorder of some of the organic groups.

PROBLEM: Check Calcd Resid. Dens. 1.00Ang From W1 3.81 eA-3
RESPONSE: This is due to limited crystal quality.

PROBLEM: High 'MainMol' Ueq as Compared to Neighbors of C22 Check
RESPONSE: This is due to disorder of some of the organic groups.

PROBLEM: Low 'MainMol' Ueq as Compared to Neighbors of K3 Check
RESPONSE: This is due to disorder of some of the counterion complexes.

PROBLEM: Low Bond Precision on C-C Bonds .... 0.05037 Ang.
RESPONSE: This is due to limited crystal quality.

PROBLEM: Short C(sp3)-C(sp3) Bond C18 - C19 . 1.33 Ang.
RESPONSE: This is due to disorder of some of the organic groups that could not be modelled in a better way without restraints.

Compound $[K(crypt\text{-}222)]_3[\mu{:}\eta^3\text{-}Bi_3\{W(CO)_3\}_2]\cdot en\cdot tol$ ($[K(crypt\text{-}222)]_3$3$\cdot$en$\cdot$tol)

PROBLEM: Isolated Metal Atom found in Structure (Unusual) Bi2 Check
RESPONSE: This is a software error that does not account for the given Bi-Bi and Bi-W bond lengths.

PROBLEM: Short C(sp3)-C(sp3) Bond C27 - C28 . 1.26 Ang.
RESPONSE: This is due to disorder of some of the organic groups that could

not be modelled in a better way without restraints.
PROBLEM: Check Calcd Resid. Dens. 0.92Ang From C4 2.53 eA-3
RESPONSE: This is due to limited crystal quality.

Compound $[K(crypt\text{-}222)]_3[\mu^2{:}\eta^3\text{-}Bi_3\{W(CO)_3W(CO)_4\}]\cdot 3en\cdot tol$ ($[K(crypt\text{-}222)]_3$4$\cdot$3en$\cdot$tol)

PROBLEM: Single Bonded Metal Atom in Structure (Unusual) Bi2 Check
RESPONSE: This is a software error that does not account for the given Bi-Bi and Bi-W bond lengths.

PROBLEM: Low Bond Precision on C-C Bonds .... 0.02554 Ang.
RESPONSE: This is due to limited crystal quality.

PROBLEM: Check Calcd Resid. Dens. 1.05Ang From W2 3.27 eA-3
RESPONSE: This is due to limited crystal quality.

PROBLEM: Check Calcd Resid. Dens. 1.94Ang From Bi1 -2.56 eA-3
RESPONSE: This is due to limited crystal quality.

Compound [K(crypt)]$_2$[W$_2$CO$_8$(μ-H$_2$)]·0.5tol ([K(crypt-222)]$_2$5·0.5tol)

PROBLEM: U(iso) H Smaller than U(eq) W1 by 0.055 Ang**2
RESPONSE: This is due to limited crystal quality.

PROBLEM: Low Bond Precision on C-C Bonds .... 0.02467 Ang.
RESPONSE: This is due to limited crystal quality.

5. Infrared Spectroscopy

Supplementary Figure 27 shows the ATR-infrared spectrum of the ground crystals obtained of the standard reaction (reaction time = 2 h). The calculated vibrational frequencies for the calculated anions are highlighted in colours: [**1**^{calc}]$^{3-}$ = grey, [**2**^{calc}]$^{2-}$ = green, [**3**^{calc}]$^{3-}$ = yellow, [**4**^{calc}]$^{3-}$ = orange and [**5**^{calc}]$^{2-}$ = blue.

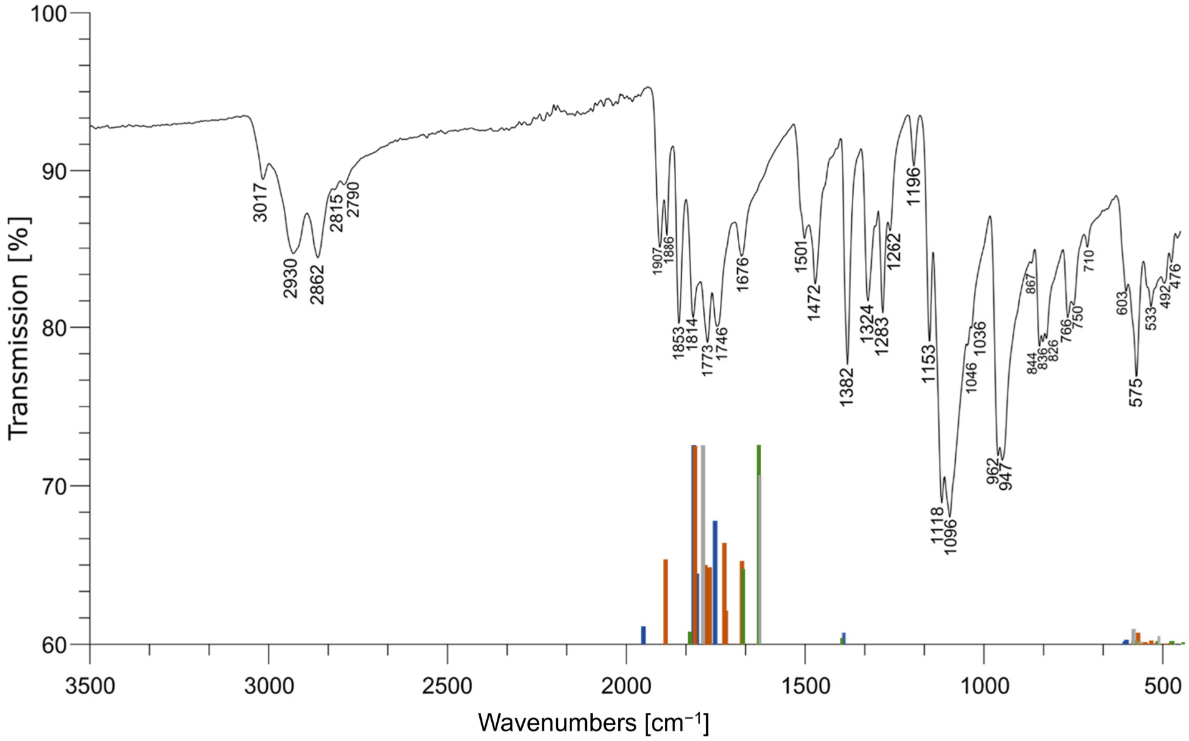

Supplementary Figure 27. Infrared Spectrum of the ground crystals obtained from reaction at room temperature for two hours. The recorded spectrum is shown in black; the calculated vibrational frequencies are coloured as [**1**^{calc}]$^{3-}$ = grey, [**2**^{calc}]$^{2-}$ = green, [**3**^{calc}]$^{3-}$ = yellow, [**4**^{calc}]$^{3-}$ = orange and [**5**^{calc}]$^{2-}$ = blue. The separate calculated spectra are additionally given in **Supplementary Figures 28 – 32**.

6. Supplementary Information on the Quantum Chemical Investigations

6.1 Optimised Molecules, Atoms, and Anions

Supplementary Table 9 shows all molecules, atoms and anions optimized to calculate the reaction pathways.

Supplementary Table 9. Overview of calculated optimised molecules, atoms, and anions used to generate a proposal for the reaction paths. The energies are given in Hartree. The atoms are shown in the following colours: Bi = dark blue, Ga = pink, C = grey, W= light blue, O = red, H = white.

$[\mathbf{1}^{calc}]^{3-}$	$[\mathbf{2}^{calc}]^{2-}$	$[\mathbf{3}^{calc}]^{3-}$
−1052.013159501 H	−522.6296475793 H	−1459.868831442 H
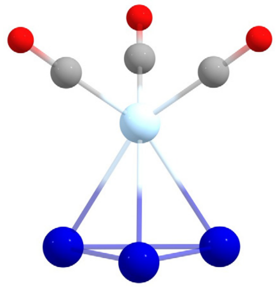	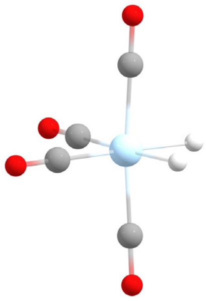	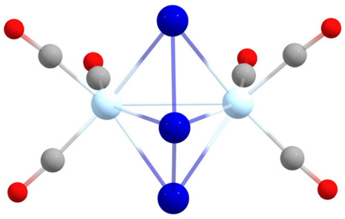
**$[\mathbf{4}^{calc}]^{3-}$**	**$[\mathbf{5}^{calc}]^{2-}$**	**$(GaBi_3)^{2-}$**
−1573.272546826 H	−1043.889807281 H	−2568.96081944 H
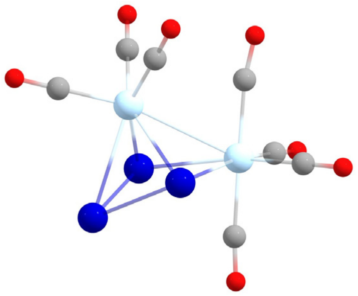	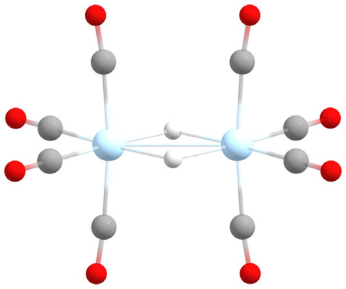	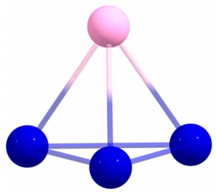
**$[W(cod)(CO)_4]$**	**CO**	**Ga^0**
−833.4269061415 H	−133.3754743511 H	−1924.793243303 H
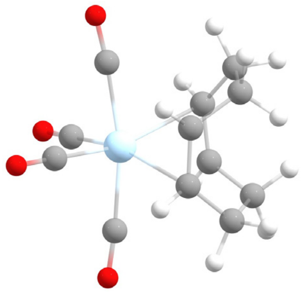	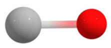	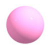

Supplementary Table 9 (continued).

**cod**	**$(Bi_4)^{2-}$**	**$(Bi_3)^{2-}$**
−312.1984499725 H	−858.6894994718 H	−644.04234398808 H
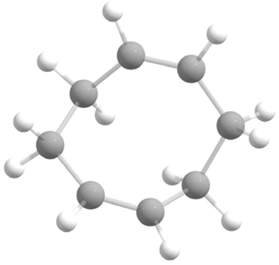	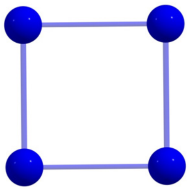	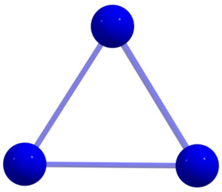
**Bi^0**	**$(Bi_2)^{2-}$**	**$(Bi_3)^{3-}$**
−214.5453299639 H	−429.4079680938 H	−644.13442969447 H
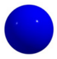	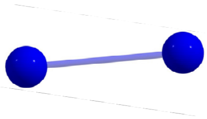	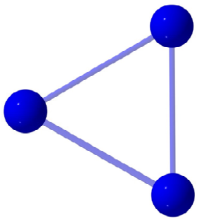
**$C_2N_2H_8$ (en)**	**$(C_2N_2H_7)^-$ (en$^-$)**	**H_2**
−190.63263967469 H	−190.08618377915 H	−1.1800544673 H
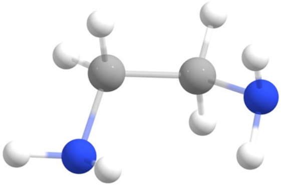	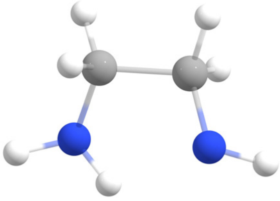	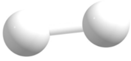
**$[W_2(CO_8)]^{2-}$**	**$[W(CO)_4]^{2-}$**	**$[W(CO)_4]^-$**
−1042.612182564 H	−521.3833239226 H	−521.3833239226 H
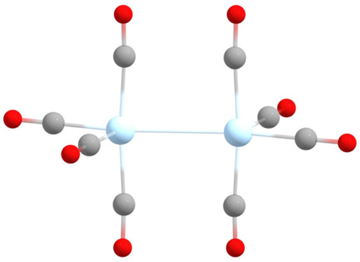	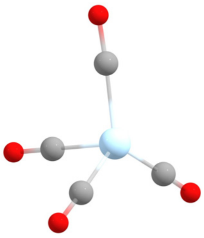	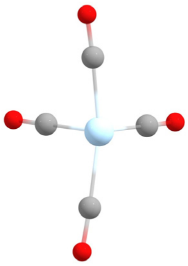

6.2 Calculated Vibrational Spectra

Supplementary Figures 28 – 32 show the calculated vibrational spectra of anions [**1**^{calc}]$^{3-}$, [**2**^{calc}]$^{2-}$, [**3**^{calc}]$^{3-}$, [**4**^{calc}]$^{3-}$, and [**5**^{calc}]$^{2-}$, respectively.

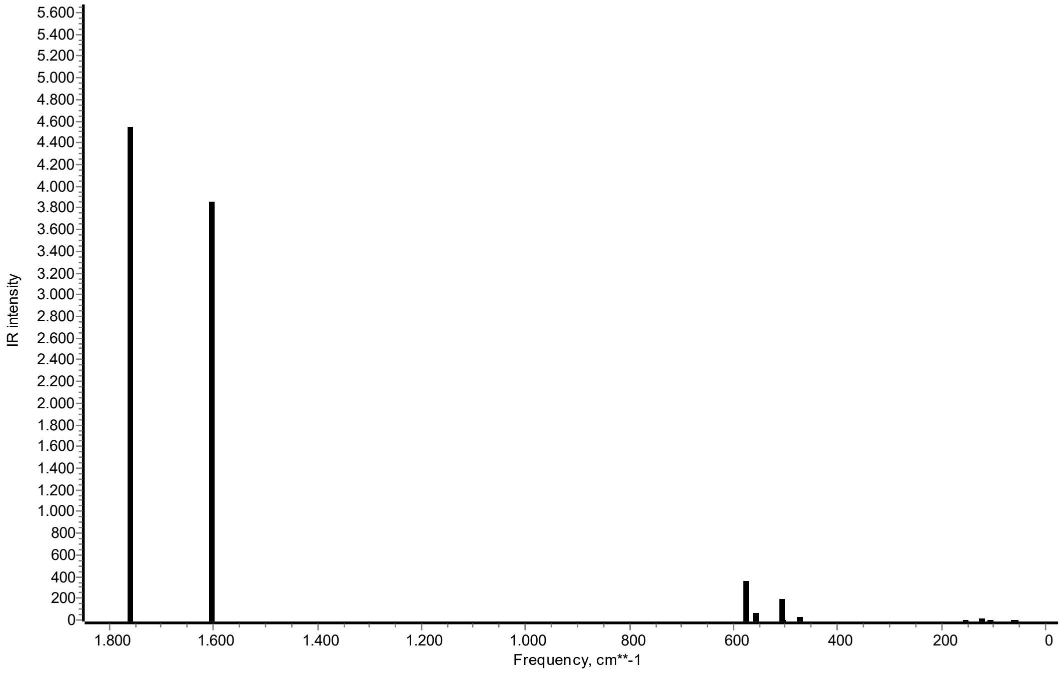

Supplementary Figure 28. Calculated vibrational spectrum of [1^{calc}]$^{3-}$.

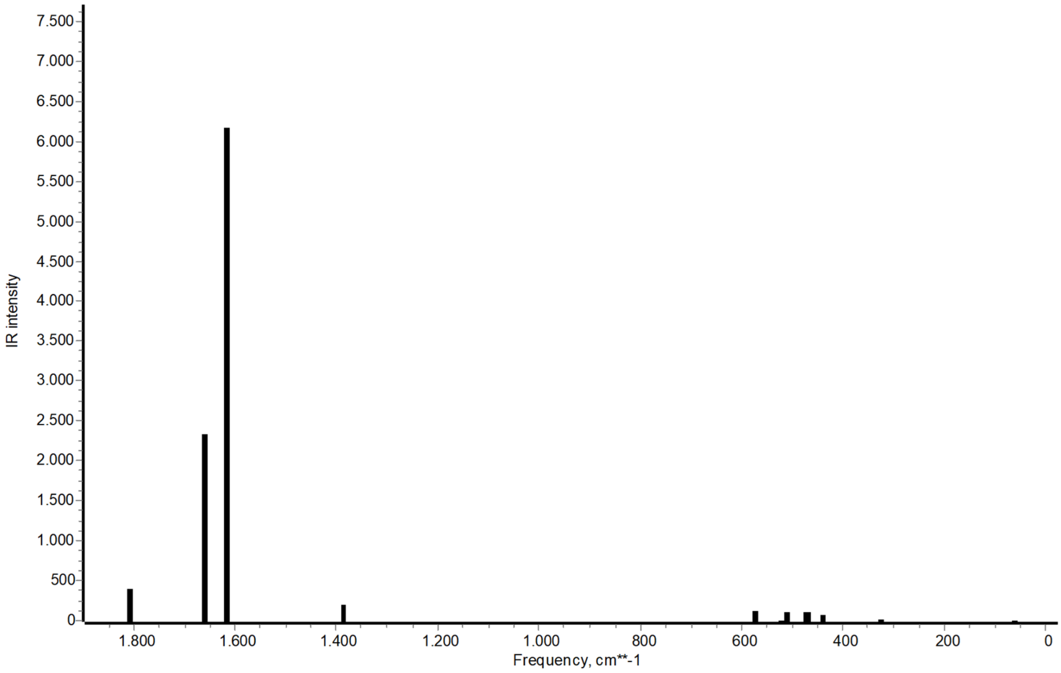

Supplementary Figure 29. Calculated vibrational spectrum of [2^{calc}]$^{2-}$.

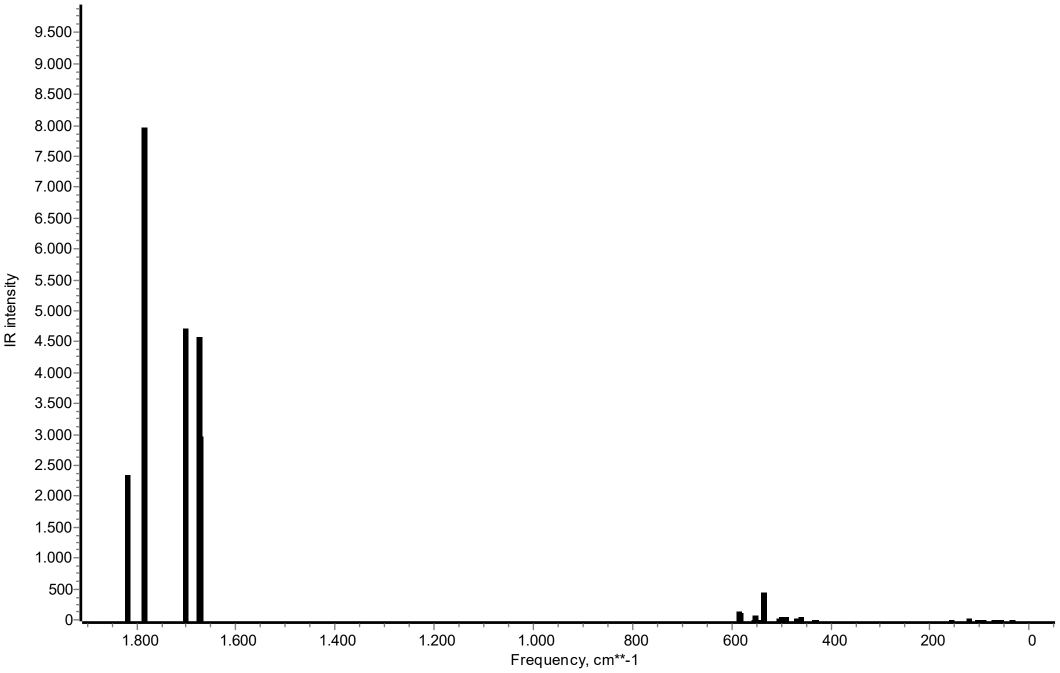

Supplementary Figure 30. Calculated vibrational spectrum of $[3^{calc}]^{3-}$.

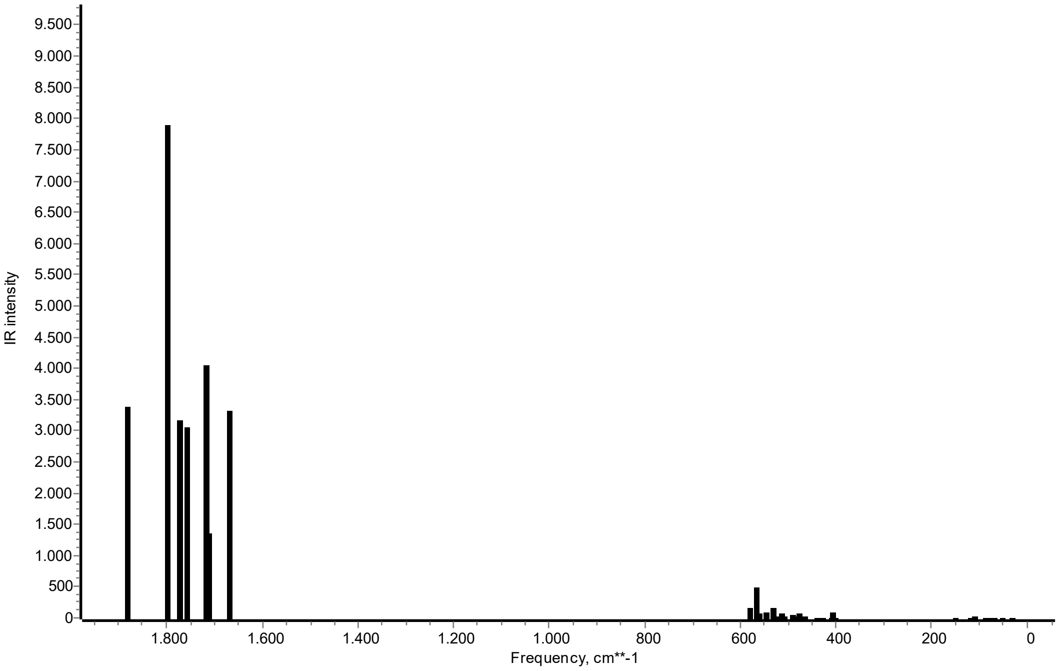

Supplementary Figure 31. Calculated vibrational spectrum of $[4^{calc}]^{3-}$.

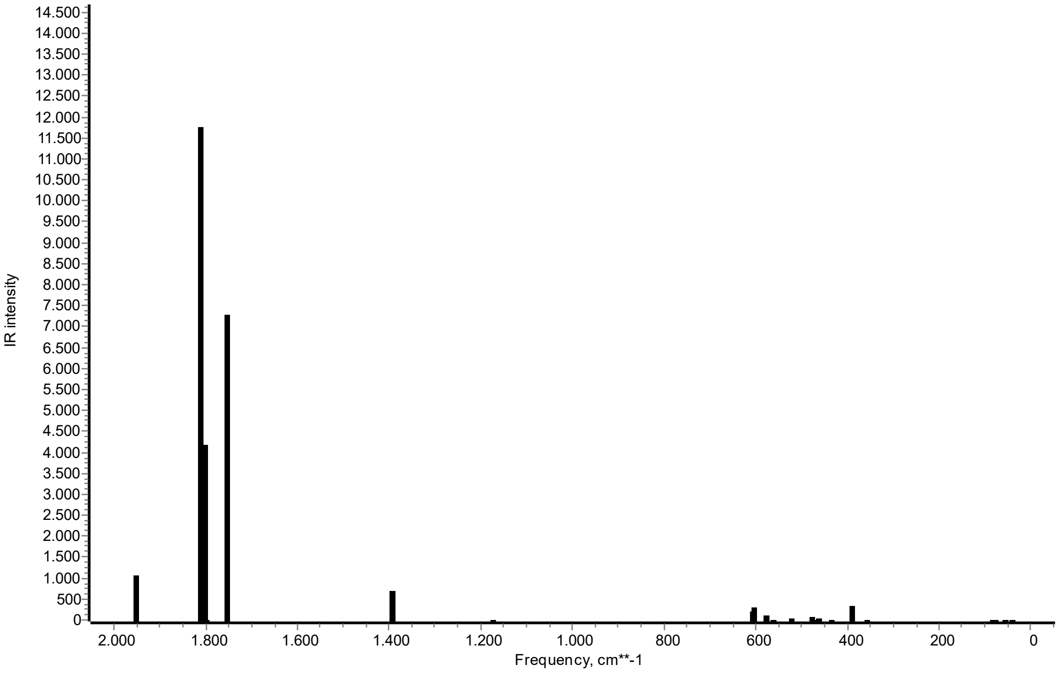

Supplementary Figure 32. Calculated vibrational spectrum of [5^{calc}]$^{2-}$.

7. Supplementary Information on the Reaction Pathways

7.1 Supplementary Calculated Reaction Pathways and Corresponding Reaction Energies

Supplementary Figure 33 summarises calculated reactions that were considered to model the preliminary steps to form small polybismuthide anions.

Preliminary step describing the formation of (open-shell) Bi_3^{2-} from $(GaBi_3)^{2-}$

$(GaBi_3)^{2-} \longrightarrow Ga^0 + Bi_3^{2-}$ $\Delta E = +329\ kJ\ mol^{-1}$

Preliminary steps describing the formation of (closed-shell) Bi_2^{2-}, Bi_3^{3-}, Bi_4^{2-} from (open-shell) Bi_3^{2-}

$2\ Bi_3^{2-} \longrightarrow 0.5\ Bi_4^{2-} + Bi_3^{3-} + Bi^0$ $\Delta E = +158\ kJ\ mol^{-1}$

$3\ Bi_3^{2-} \longrightarrow Bi_4^{2-} + 2\ Bi_2^{2-} + Bi^0$ $\Delta E = +200\ kJ\ mol^{-1}$

$2\ Bi_3^{2-} \longrightarrow Bi_3^{3-} + 0.5\ Bi_2^{2-} + 2\ Bi^0$ $\Delta E = +409\ kJ\ mol^{-1}$

$2\ Bi_3^{2-} \longrightarrow 3\ Bi_2^{2-}$ $\Delta E = -366\ kJ\ mol^{-1}$

Supplementary Figure 33. Calculated preliminary reaction steps and corresponding reaction energies.

Supplementary Figure 34 summarises calculated alternative steps to form the anions [**1**^{calc}]$^{3-}$, [**2**^{calc}]$^{2-}$, [**3**^{calc}]$^{3-}$, [**4**^{calc}]$^{3-}$, and [**5**^{calc}]$^{2-}$ considering Bi_3^{3-} or Bi_2^{2-} as precursor units. Note that none of the alternatives yielded a series of only exoenergetic reaction steps.

Considering Bi_3^{3-}

$[W(cod)(CO)_4] + 4\ Bi_3^{3-} \longrightarrow \underset{[\mathbf{1}^{calc}]^{3-}}{[Bi_3W(CO)_3]^{3-}} + 4.5\ Bi_2^{2-} + CO\uparrow + cod$ $\Delta E = +109\ kJ\ mol^{-1}$

$[W(cod)(CO)_4] + 4\ Bi_3^{3-} + 2\ en \longrightarrow \underset{[\mathbf{2}^{calc}]^{2-}}{[W(CO)_4H_2]^{2-}} + 2\ Bi_4^{2-} + 2\ Bi_2^{2-} + cod + 2\ en^-$ $\Delta E = -91\ kJ\ mol^{-1}$

Considering Bi_3^{2-}

$[W(cod)(CO)_4] + 4\ Bi_3^{2-} \longrightarrow \underset{[\mathbf{1}^{calc}]^{3-}}{[Bi_3W(CO)_3]^{3-}} + 2\ Bi_4^{2-} + 0.5\ Bi_2^{2-} + CO\uparrow + cod$ $\Delta E = +194\ kJ\ mol^{-1}$

$[W(cod)(CO)_4] + 4\ Bi_3^{2-} + 2\ en \longrightarrow \underset{[\mathbf{2}^{calc}]^{2-}}{[W(CO)_4H_2]^{2-}} + 2\ Bi_4^{2-} + 4\ Bi^0 + cod + 2\ en^-$ $\Delta E = -493\ kJ\ mol^{-1}$

Supplementary Figure 34. Calculated alternative reaction steps and corresponding reaction energies.
