# Supplementary material for: Insight into the formation of bismuth-tungsten carbonyl clusters
Source: Commun Chem. 2023 Jun 5;6:109. doi: 10.1038/s42004-023-00905-6 (PMC10241888; doi:10.1038/s42004-023-00905-6)
Supplement: Supplementary file 2 — Description of Additional Supplementary Files [file 42004_2023_905_MOESM2_ESM.pdf]

Description of Additional Supplementary Files

File name: Supplementary Data 1

Description: CIF for the crystal structure of the compound comprising anion 1

File name: Supplementary Data 2

Description: CIF for the crystal structure of the compound comprising anion 2

File name: Supplementary Data 3

Description: CIF for the crystal structure of the compound comprising anion 3

File name: Supplementary Data 4

Description: CIF for the crystal structure of the compound comprising anion 4

File name: Supplementary Data 5

Description: CIF for the crystal structure of the compound comprising anion 5

File name: Supplementary Data 6

Description: Summary of Cartesian coordinates and total energies of all molecules computed using quantum chemical (DFT) methods
